# Precise Mechanochemical Scission of DNA Guided by Secondary Structures

**DOI:** 10.1021/jacs.5c20882

**Published:** 2026-03-25

**Authors:** Johannes Hahmann, Arjuna Selvakumar, Boris N. Schüpp, Montgomery Labudda, Yuanxu Zhou, Gurudas Chakraborty, Frauke Gräter, Andreas Herrmann

**Affiliations:** † Institute of Technical and Macromolecular Chemistry, 9165 RWTH Aachen University, Worringerweg 2, 52074 Aachen, Germany; ‡ 38813 DWI − Leibniz-Institute for Interactive Materials, Forckenbeckstraße 50, 52074 Aachen, Germany; § Max Planck School Matter to Life; ∥ 28308 Max Planck Institute for Polymer Research, Ackermannweg 10, 55128 Mainz, Germany

## Abstract

DNA has recently been exploited as a biopolymer, enabling sequence-specific, mechanophore-free mechanochemistry with few-nucleotide-precision scission and monomer-level analysis of breakage. Strategically positioned nicks in double-stranded DNA (dsDNA) have previously been shown to act as mechanophores, concentrating force on the non-nicked strand opposite each nick to induce localized scission; however, base fraying inherently limits this precision. To address this, we identify, for the first time, the potential of DNA hairpins to serve as ‘biomechanophores’ and direct precise mechanochemical scission of DNA. Using a dsDNA construct containing a centrally embedded hairpin motif, we show that ultrasonication induces cleavage regioselectively opposite the hairpin loop. Force localization around nucleotides on the nonhairpin strand was rationalized by all-atom, constant-force molecular dynamics (MD) simulations. Next-generation sequencing (NGS) of sonicated fragments revealed a narrow, well-defined scission distribution on the nonhairpin strand, in contrast to a much broader, bimodal-like distribution on the hairpin strand. Together with the simulations, these results support a two-step cleavage mechanism. Our findings establish DNA hairpins as the first functional biomechanophores capable of directing mechanically induced strand scission with high spatial precision, expanding the mechanochemical toolbox for nucleic acid manipulation.

## Graphical abstract

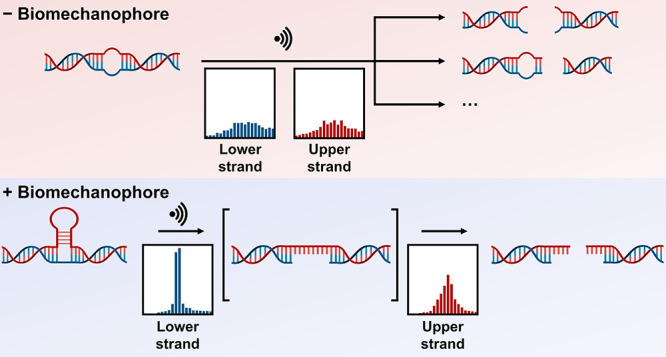

Polymer mechanochemistry harnesses mechanophores—force-responsive molecular units embedded within synthetic polymers—to drive force-induced transformations at the molecular level.^1^ This transformation of mechanical stress into chemical or optical signals offers wide-ranging applications, including mechanocatalysis,^2–4^ visual stress detection,^5–8^ and the development of self-healing^9,10^ and self-immolative^11,12^ materials. Despite their advantages, conventional mechanophores face challenges such as complex design requirements for bond scission under mechanical stress, tedious synthesis, and the difficult analysis of their bond-breaking mechanics.^13^ These constraints underscore the need for mechanophore platforms that allow precise, molecularly defined investigations of mechanical scission. In this context, DNA secondary structures offer a uniquely powerful biopolymer-based model system, owing to their unparalleled sequence controllability, structural polymorphism, and programmable functionality. While mechanophores in the form of specific dinucleotide motifs have been explored in native dsDNA,^14^ the mechanophore-like behavior of DNA secondary structures has, to date, remained unexplored. Recently, we demonstrated mechanophore-free mechanochemistry of DNA by inducing site-specific DNA cleavage through the strategic introduction of nicks.^15^ Postscission analysis using next-generation sequencing (NGS) revealed a high degree of regioselectivity; however, the base fraying mechanism inherently limited cleavage precision. To address this, we reveal the potential of DNA hairpins, an alternative B-form DNA secondary structure, to act as ‘biomechanophores’ by guiding precise mechanochemical scission of DNA. We designed a ∼ 750 base pair (bp) double-stranded DNA (dsDNA) construct incorporating a central hairpin motif *via* a ligation-based strategy (Scheme 1). To this end, we first amplified the left and right fragments *via* polymerase chain reaction (PCR). These fragments were then digested to generate specific, nonpalindromic sticky ends, enabling the ligation of various short middle fragments containing the hairpin motif. This approach allowed systematic evaluation of loop size (L) and stem length (S) variations on one of the strands (hairpin strand), as well as unpaired nucleotides (N) positioned opposite the hairpin on the other strand (nonhairpin strand). We denote the resulting secondary structures as Hp­(N, S, L). Importantly, when S = 0, the structure contains a bulge in which unpaired nucleotides on the hairpin strand are opposite unpaired nucleotides on the nonhairpin strand. For example, for Hp­(4,0,4), there is a symmetric bulge with four unpaired nucleotides opposite each other. Of note, we first used the well-known DNA hairpin motif whose X-ray structure was originally reported by Chattopadhyaya et al.^16^ We hypothesized a two-step rupture process for these hairpin structures upon ultrasonication, arising from force focusing due to the orthogonal orientation of the hairpin relative to the applied shear: a precise cleavage at the hairpin-opposing junction followed by a less-precise, but still highly localized scission on the hairpin-containing strand. We tested this hypothesis in a combined experimental and computational approach (Scheme 1). We determined distributions of fragment sizes with monomer-level precision using NGS,^15^ and used all-atom constant-force molecular dynamics (MD) simulations to reveal the force concentration along the two DNA strands prior to their respective scission, in order to explain variations in scission specificity across the tested structures (Scheme 1).

**Scheme 1:**
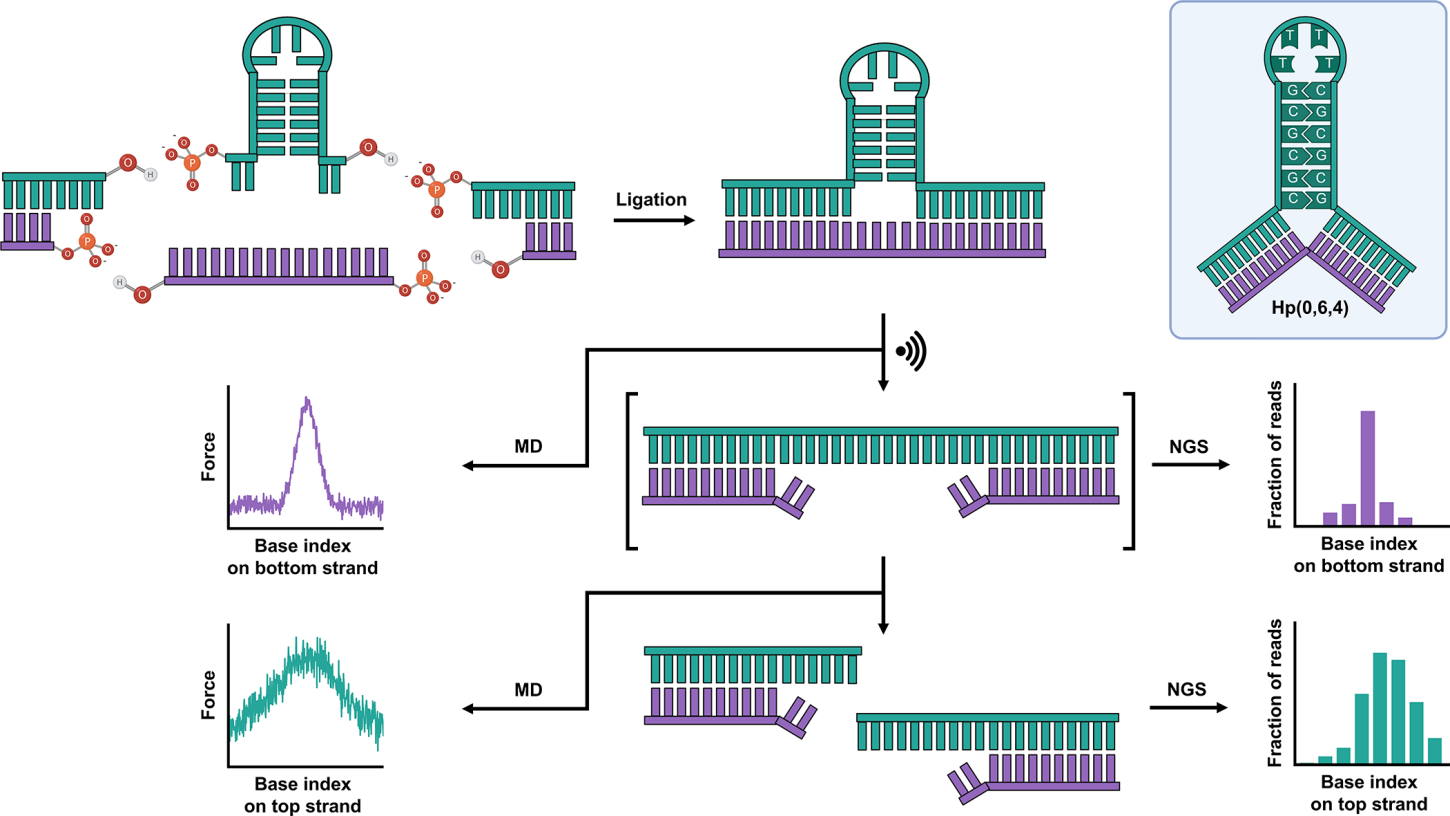
Schematic of the Ligation-Based DNA Construct Design and Downstream Processes^a^

a Structures are denoted as Hp­(N, S, L), where L indicates the loop size and S the stem length on the hairpin strand, and N denotes the number of unpaired nucleotides on the nonhairpin strand. The downstream process involves ultrasonication of the ligated product, followed by characterization by gel electrophoresis. Scission events are subsequently analyzed with monomer-level precision using NGS.^15^ Mechanistic interpretations are derived from the NGS data in combination with all-atom, constant-force MD simulations.

We started with sonicating Hp­(0,6,4) for 8 min at 20 kHz (Figure 1a). This resulted in a sharp band at half the size of the original dsDNA (Figure 1b). To further evaluate this cleavage event at nucleotide-level resolution, we conducted Illumina sequencing using the same procedure described previously,^15^ enabling precise determination of the ultrasonication-induced scission points on the nonhairpin (Figure 1c) and hairpin strands (Figure 1d).

**Figure 1:**
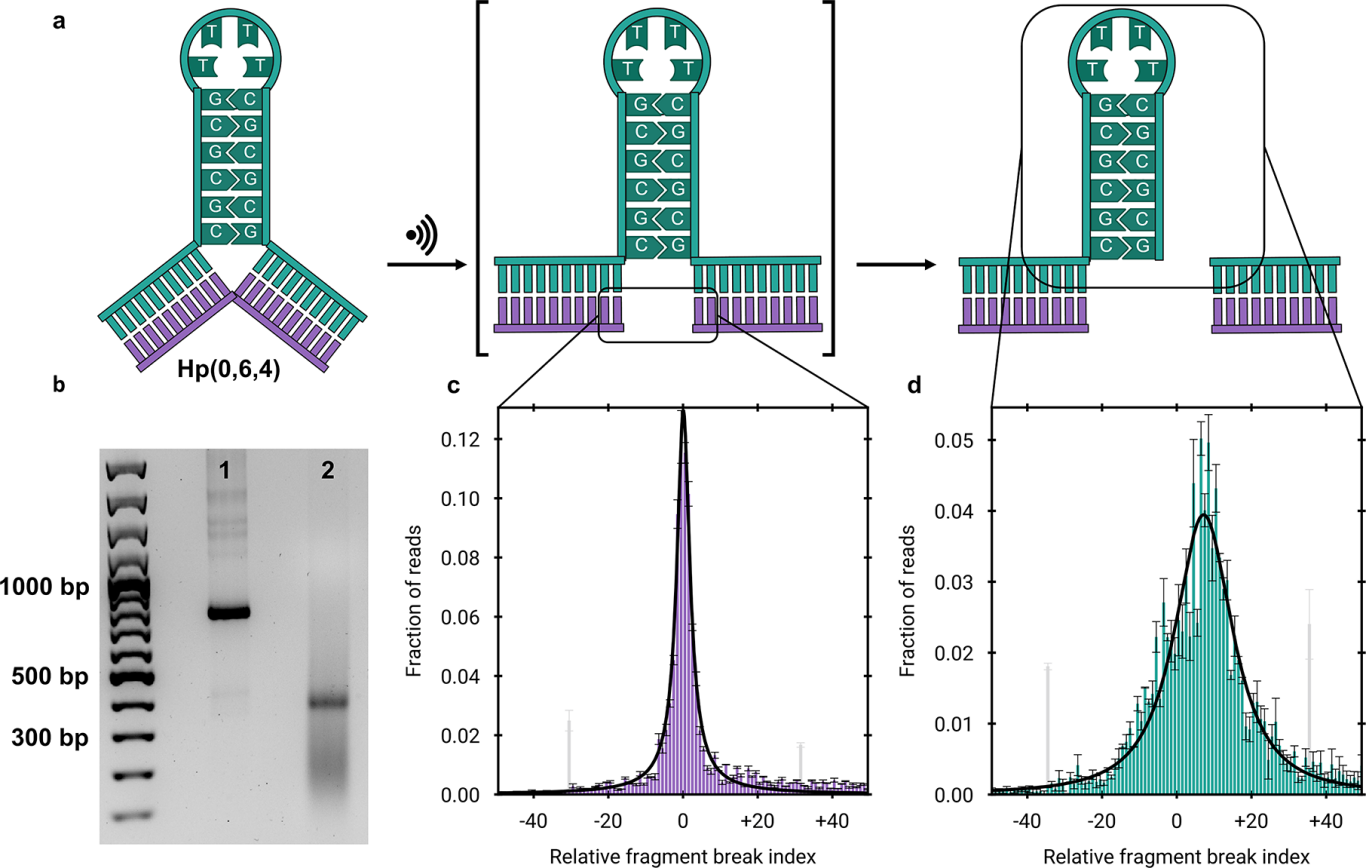
Mechanochemical scission of Hp­(0,6,4) demonstrated by agarose gel electrophoresis and NGS. a) Schematic of the hypothesized mechanism for the scission of the central secondary structure within a 750 bp dsDNA. b) Agarose gel electrophoresis of the investigated system shown in a). Lane 1: untreated Hp­(0,6,4); lane 2: Hp­(0,6,4) after 8 min of sonication. A sharp band is visible at ∼ 400 bp, indicating precise central scission. All sonication product in lane 2, that migrated faster than the reactant, was excised from the gel. c) NGS-derived scission distribution of the nonhairpin strand (purple), yielding an interquartile range of 5.4 ± 0.3 nt. d) NGS-derived scission distribution of the hairpin strand (green), indicating less precise scission. Grayed out bars indicate ligation artifacts that have been replaced by the average signal of their three-nucleotide neighborhood for the fitting procedure.

A narrow scission distribution was observed for the nonhairpin strand, whereas the hairpin strand exhibited a significantly broader distribution. Notably, both strands showed minor signals in the order of 2–5% of the total reads, resembling “antennas”, that did not align with the overall breaking pattern, appearing approximately ± 30 nucleotides away (shown greyed out). These artifacts likely result from incomplete ligation during construct synthesis, as T4 DNA ligase does not achieve full conversion under the reaction conditions.^17^ To verify this, we assembled an identical construct by enzymatically digesting two dsDNA fragments with λ-exonuclease to generate two long ssDNAs, which were then rehybridized (Figure S2a). Sonication of this new construct yielded scission distributions identical to those of the original construct but without the artifactual “antenna” signals (Figure S2b,c). In order to obtain unbiased fits of the scission distributions, these signals have been replaced with the average of the neighboring signals (three nucleotides in both directions). The width of the scission distribution with 5.4 ± 0.3 nt on the nonhairpin strand is up to almost twice as narrow as the one previously reported for the nicked DNA system^15^ (Table S11 and Figure 3e). This gain in precision is particularly notable, given that the hairpin structures – unlike nicked DNA – require two successive scission events on opposite backbones. In this case, the behavior of our construct as a biomechanophore exclusively stems from the formation of secondary structures.

To investigate the proposed two-step cleavage mechanism, we performed fully atomistic, constant-force MD simulations in two stages. First, a 2.0 nN force was applied to the intact hairpin to generate a force profile of the relevant backbone bonds. Second, the central residue on the nonhairpin strand was precleaved, and the resulting structure was simulated under the same 2.0 nN force. With this procedure, the two-step rupture process is treated as a model assumption, allowing us to probe a plausible mechanistic pathway using an effective force-based proxy for ultrasonic scission. Structural analysis (Figure 2a) shows that the hairpin remains largely intact during the first step, but fully unfolds in the second step, exposing a single-stranded bridge. Time-resolved end-to-end distances (Figure 2b) at lower forces (0.1 nN) reveal two major events associated with unfolding: an initial relaxation of the junction at the start of the simulation, followed by loss of hairpin base pairing after ∼ 30 ns. Subsequent increases in end-to-end distance arise from bond stretching and a transition to zip-DNA, an extended ladder-like conformation.^18^ At higher forces, these steps occur simultaneously (Figure S13). We consider the C3′–O3′ bond to be most relevant for ultrasound-induced scission^14,19^ and compute time-averaged forces during both scission steps; although the precise electronic nature of the rupture (homolytic versus heterolytic) is unresolved, force provides a pathway-agnostic measure of mechanical susceptibility. Prior to the first scission (Figure 2c), the highest forces are localized near the hairpin junction on the nonhairpin strand, consistent with the precise scission observed experimentally for that strand. Prior to the second scission, the force is transmitted solely through the single-stranded bridge, creating a broad region of elevated forces on the hairpin strand within residues that formerly participated in base pairing.

**Figure 2:**
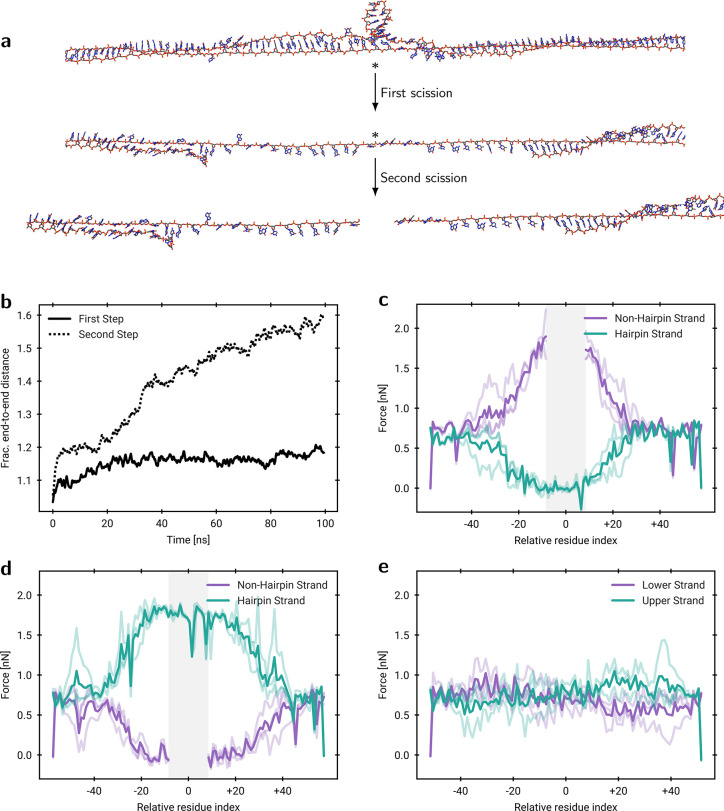
Two-step mechanism of DNA hairpin cleavage in MD simulations. a) Representative MD snapshots under load show the two sequential scission steps, with resulting fragments obtained after the second cleavage. Stars denote the most probable break points identified from force analysis. b) Time evolution of the fractional end-to-end distance (running average over 50 frames) at 0.1 nN, indicating hairpin unfolding during the second scission step. c) Time-averaged force on the C3′–O3′ bond per residue for the Hp­(0,6,4) construct before the first scission under a constant 2.0 nN force, revealing higher forces on the nonhairpin strand. The gray bar indicates residue indices, *i.e.* nucleotides present only in the hairpin region. d) Time-averaged C3′–O3′ bond force per residue for Hp­(0,6,4) before the second scission under a constant 2.0 nN force, showing higher forces on the hairpin strand. The gray bar indicates residue indices present only in the hairpin. e) Time-averaged C3′–O3′ bond force for the control system Hp­(4,0,4), which contains a symmetric bulge of four unpaired nucleotides on opposite strands, before the first scission, showing no strand-specific force enrichment.

Notably, this force-enriched region is much wider than in the first step, explaining the broader breakage distribution observed experimentally for this strand. In the control system Hp­(4,0,4), which lacks strand asymmetry, no significant accumulation of force is observed on either strand or region (Figure 2e), consistent with its scission distributions (Figure S5). Thus, the MD simulations are in line with the experimental results and further support the proposed mechanism.

To further elucidate the secondary structural features responsible for precise scission (Figure 1) and force concentration on the nonhairpin strand (Figure 2), we examined a hairpin variant lacking a stem, designated Hp­(0,0,4). We hypothesized that if stem formation is critical for effective force transduction and localized scission, its absence would significantly reduce cleavage precision. Consistent with this hypothesis, sonication of Hp­(0,0,4) resulted in a markedly broader scission distribution compared to constructs containing even minimal stem regions (Figure 3b,c). This reduction of distribution width by more than half through the inclusion of just four base pairs in the stem highlights the critical role of stem stability in mechanical focusing.

**Figure 3:**
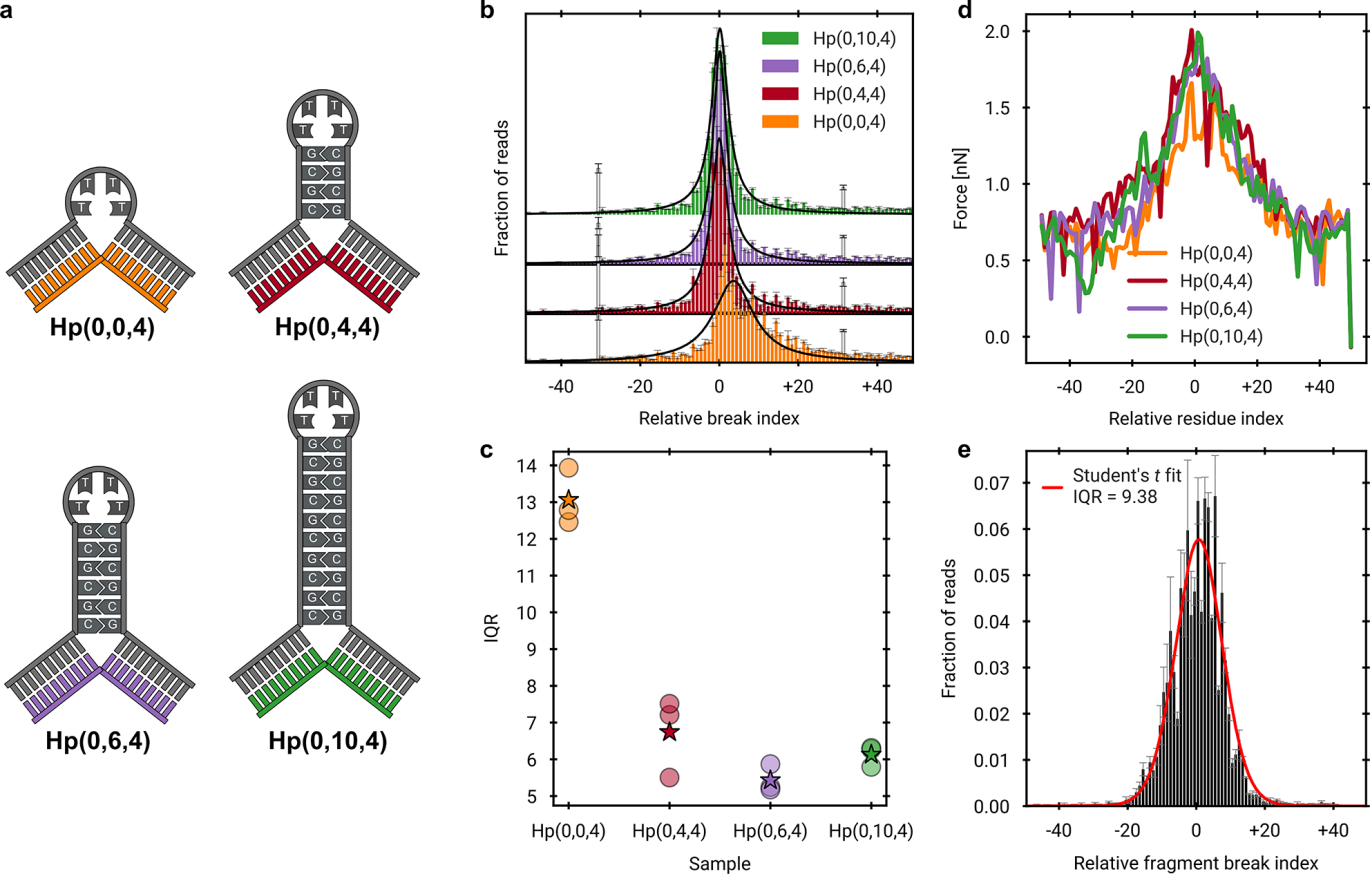
Inclusion of a stem is crucial for precise scission. a) Schematic of the central secondary structure motifs employed within a 750 bp dsDNA. b) Scission distributions of the nonhairpin strand obtained by NGS, fitted with a Student’s t-fit to extract the interquartile range (IQR) as a parameter of width. c) IQR values for the scission distributions. Hp­(0,0,4) exhibits a notably, wider IQR than the other constructs, indicating less precise scission. d) Time-averaged force on the C3′–O3′ bond per residue for systems with varying hairpin stem lengths, evaluated prior to the first scission under a constant 2.0 nN load. The time-average was averaged again over three independent simulations. Distributions are broadly similar, with Hp­(0,0,4) showing a distinguishable deviation. e) Experimental scission distribution on the non-nicked strand of a previously published nicked DNA system.^15^ The IQR is larger than for the hairpin structures (except Hp­(0,0,4)), consistent with reduced scission precision.

To further support our experimental observations, we performed constant-force MD simulations on the same motif series and analyzed the time-averaged force on the C3′–O3′ bonds of the nonhairpin strand prior to the first scission (Figure 3d). Across all systems, forces peak at or near the hairpin junction, indicating precise scission opposite the hairpin. Hp­(0,0,4) exhibits a distinctly flatter distribution with a lower maximum, consistent with the less precise scission observed experimentally. Further MD simulations in which the base composition of the hairpin stem was changed from GC base pairs to AT base pairs show that the shape of the force distributions is largely independent of the stem sequence (see Figures S18 and S19). These results suggest that structural asymmetry between strands – even in the absence of a hairpin – can induce localized scission; however, the highest precision is achieved when force transmission at the junction is tightened by incorporating a hairpin stem of at least four base pairs. Comparison to prior results on nicked DNA structures,^15^ which require a single precise break to produce overall specific fragmentation (Figure 3e), indicates that introducing a hairpin affords additional control over the exact breaking position.

In summary, we show that DNA hairpins function as mechanochemically active motifs that localize stress and direct strand scission with few-nucleotide precision. NGS and all-atom MD simulations together reveal a two-step cleavage mechanism in which forces concentrate in the nonhairpin strand opposite the hairpin loop and are tuned primarily by stem length. We expect that the exact hairpin sequence can influence the shape of the scission distribution, although this effect is likely to be quantitatively smaller than structural parameters such as the presence or absence of a stem. These findings establish hairpins as programmable biomechanophores, a previously unrecognized, sequence-defined design principle. We further anticipate that this concept is not limited to simple hairpin motifs and can be extended to more complex DNA secondary structures that focus mechanical stress, such as multiloop hairpins or G-quadruplexes, providing a general framework for mechanochemical control in nucleic acids and beyond.

## Supplementary Material



## Notes

Experimental methods, sequencing analysis, and molecular dynamics simulations (PDF)
