# Supplementary material for: Precise Mechanochemical Scission of DNA Guided by Secondary Structures
Source: J Am Chem Soc. 2026 Mar 25;148(13):13494–9. doi: 10.1021/jacs.5c20882 (PMC13067279; doi:10.1021/jacs.5c20882)
Supplement: Supplementary file 1 [file ja5c20882_si_001.pdf]

Supporting Information

Precise Mechanochemical Scission of DNA Guided by Secondary Structures

Johannes Hahmann[‖], Arjuna Selvakumar[‖], Boris N. Schüpp[‖], Montgomery Labudda,
Yuanxu Zhou, Gurudas Chakraborty[*], Frauke Gräter[*], and Andreas Herrmann[*]

[‖]These authors contributed equally to this work.

*Corresponding authors.
Email: chakraborty@dwi.rwth-aachen.de; graeter@mpip-mainz.mpg.de; herrmann@dwi.rwth-aachen.de

Contents

1 General information **3**

2 Methods **3**
2.1 Generation of DNA with central hairpin secondary structure 3
2.1.1 Generation of ligation substrates 3
2.1.2 Ligation reaction 4
2.1.3 Generation of long ssDNA substrates by Lambda Exonuclease digestion 5
2.1.4 Hybridization to the central hairpin-containing product 6
2.2 Ultrasound-induced scission of DNA 6
2.3 Capillary Electrophoresis 6
2.4 Illumina Sequencing 6
2.4.1 Experimental Procedure 6
2.4.2 Data Analysis 7
2.5 MD Simulations 7

3 Supporting Results **9**
3.1 Capillary Electrophoresis 9
3.2 NGS Analysis 9
3.2.1 Validation of Filtering Algorithm 9
3.2.2 Effect of Ligation Strategy 10
3.2.3 Comparison to dsDNA 10
3.2.4 Filtering Accuracy and Read Quality 12
3.2.5 Breaking Distributions and Distribution Analysis 13
3.3 MD Simulations 18
3.3.1 End-to-End Distances 18
3.3.2 Forces on Different Bond Types 20
3.3.3 Variation of Applied Forces 21
3.3.4 Additional Force Distributions 21
3.3.5 Variation of the Base Composition of the Stem in MD Simulations 22

4 Appendix **24**
4.1 DNA Sequences 24
4.2 Sequencing Results 26
4.3 MD Simulations 32

1 General information

The genes to generate **DNA**$_{\text{left}}$ and **DNA**$_{\text{right}}$ were purchased from Eurofins Genomics. The primers used to amplify **DNA**$_{\text{left}}$ and **DNA**$_{\text{right}}$ were acquired from Sigma-Aldrich. HPLC-purified oligonucleotides used to form the middle fragments, *i.e.* the secondary motifs, were purchased from biomers.net GmbH. All sequences for genes, primes and oligonucleotides are provided in Fig. S20-S26 and Table S13. DNA Polymerase was bought from Biovendis Products GmbH as Pfu/Psp polymerase (cat. no. TK-005222). dNTPs were purchased from GeneON GmbH (cat. no. 110-012). Restriction enzymes FastDigest BstXI (cat. no. FD1024) and FastDigest Eco31I (cat. no. FD0293), as well as T4 Ligase (cat. no. EL0012) were obtained from Thermo Scientific™. Agarose Standard (cat. no. 3810.4, Carl Roth) was used for agarose gel electrophoresis (GE), along with ROTI®GelStain Red Eco as intercalating dye (cat. no. 223C.1, Carl Roth). TAE buffer was prepared using Tris base (CAS 77-86-1, Fisher Bioreagents), acetic acid (CAS 64-19-7, Sigma) and disodium ethylenediaminetetraacetate dihydrate (EDTA) (CAS 60-00-4, Sigma) with a final concentration (1×) of 40 mM Tris, 20 mM acetic acid and 2 mM EDTA. Samples were loaded into agarose gels using TriTrack DNA Loading Dye (6×) (cat. no. R1161) and GeneRuler 100 bp plus DNA Ladder (cat. no. SM0321) both sourced from Thermo Scientific™. Gel fragments were purified with the GFX™ PCR DNA and Gel Band Purification Kit from Cytiva (cat. no. 28903471). For library preparation of sheared dsDNA, the NEBNext Ultra II DNA Library Prep Kit (cat. no. E7645L, NEB) was used. Illumina paired-end sequencing was performed on an Illumina MiSeq sequencer using MiSeq Reagent Micro Kit v2 (300-cycles) (cat. no. MS-103-1002, Illumina).

2 Methods

2.1 Generation of DNA with central hairpin secondary structure

2.1.1 Generation of ligation substrates

DNA$_{\text{left}}$ and **DNA**$_{\text{right}}$ genes were amplified *via* polymerase chain reaction (PCR). Table S1 lists the components for one PCR reaction.

Table S1: Components for PCR amplification of **DNA**$_{\text{left}}$ and **DNA**$_{\text{right}}$.

Components	V [µL]	c_{final}
PCR template	1	0.2 ng/µL
Forward primer	2.75	0.688 µM
Reverse primer	2.75	0.688 µM
dNTPs	1	0.200 mM
Pfu/Psp polymerase buffer	5.0	1×
Pfu/Psp polymerase	0.4	0.04 U/µL
Water	37.1	–
Σ	50	–

The reaction is set up in a PCR tube and then transferred to a thermocycler. In Table S2 the utilized PCR parameters are displayed.

Table S2: PCR parameters for thermocycler.

Reaction step	Cycle(s)	T [°C]	t [s]
Heat lid	–	110	∞
Initial denaturation	1	98.0	180
Denaturation	30	98.0	20
Annealing	30	66.0	30
Extension	30	72.0	60
Final extension	1	72.0	300
Storage	–	4.0	∞

The amplified **DNA**$_{\text{left}}$ and **DNA**$_{\text{right}}$ PCR products were then purified by solution purification using the GFX PCR DNA and Gel Band Purification kit (Cytiva). The purified PCR products are then restricted by two different enzymes to generate specific sticky ends. The reaction components for the restriction

digest are shown in Table S3. One fast digestion approach contains 1 μg of purified PCR product. The concentration of the purified PCR product is variable, and the volume Y is adjusted accordingly.

Table S3: Restriction digest protocol for **DNA**$_{\text{left}}$ (BstXI) and **DNA**$_{\text{right}}$ (Eco31I).

Components	V [μL]	c_{final}
PCR product (1 μg)	Y	33.3 ng/μL
FD Green Buffer	2.40	0.8×
FD Enzyme BstXI (**DNA**$_{\text{left}}$)	1.20	–
or FD Enzyme Eco31I (**DNA**$_{\text{right}}$)	1.20	–
Water	to 30	–
Σ	30	–

This mixture was incubated at 37 °C for 1 h. Subsequently, the enzyme was deactivated at 80 °C for 5 min. The solution was then purified *via* agarose gel and GFX PCR DNA and Gel Band Purification kit (Cytiva).

The central secondary motifs (Table S13) were synthesized by targeted hybridization. The reactions were set up according to Table S4 and assembled by slowly cooling from 95 °C to 4 °C with 1 °C/20 s. The targeted hybridization product was used in the subsequent ligation reaction without further purification.

Table S4: Targeted hybridization of central secondary motifs with short oligonucleotides.

Components	V [μL]	c_{final} [μM]
Non-hairpin strand	2.0	2.4
Hairpin strand	2.0	2.8
T4 Ligase buffer	0.50	1×
Water	0.50	–
Σ	5.0	–

2.1.2 Ligation reaction

In the first ligation central secondary motif is ligated to the left restriction product. The required reaction components for one reaction approach of this first ligation step are listed in Table S5. The mixture was incubated at 22 °C for 2 h and the enzyme was subsequently deactivated at 70 °C for 5 min.

Table S5: Ligation of central secondary motif with left restriction product.

Components	V [μL]	c_{final}
Central secondary motif	1.0	67.5 nM
Restriction product (left)	Y	100 nM
T4 Ligase buffer	2.0	1×
T4 DNA Ligase	1.5	0.38 U/μL
Water	to 20	–
Σ	20	–

In the second ligation the right restriction product is ligated to the first ligation product. The required reaction components for one reaction approach of second ligation are listed in Table S6.

Table S6: Second ligation step to generate full-length DNA.

Components	V [µL]	c_{final}
Restriction product (right)	Y	200 nM
T4 Ligase buffer	2.0	2×
T4 DNA Ligase	0.5	0.25 U/µL
Water	to 10	–
Σ	10	–

The reaction mixture is added directly to the first ligation reaction solution. Ligation proceeds at 8 °C for 16 h with subsequent deactivation of the enzyme at 70 °C for 5 min. To the resulting solution 3.3 µL per 30 µL 1 wt.% SDS (dilution to 0.1 wt.%) were added. Then, the solution was exposed to 60 °C for 10 min, and immediately cooled on ice. Tritrack Loading Dye (1 µL per 5 µL of sample) was quickly added, the solution was mixed thoroughly and the product was purified *via* agarose gel and GFX PCR DNA and Gel Band Purification kit (Cytiva).

2.1.3 Generation of long ssDNA substrates by Lambda Exonuclease digestion

Two dsDNA strands (Fig. S22 and S23) were initially amplified by PCR to generate substrates for Lambda Exonuclease digestion. One primer was modified with a 5'-phosphate group to enable strand-selective degradation. The components for one PCR reaction are listed in Table S7.

Table S7: Components for PCR amplification of Lambda Exonuclease substrates.

Components	V [µL]	c_{final}
PCR template	1	0.2 ng/µL
Forward primer	2	0.5 µM
Reverse primer	2	0.5 µM
Pfu Master Mix Green	25	1×
Water	10	–
Σ	50	–

The PCR reaction was assembled in a PCR tube and transferred to a thermocycler. The thermocycler parameters are shown in Table S8.

Table S8: PCR parameters for Lambda Exonuclease substrates.

Reaction step	Cycle(s)	T [°C]	t [s]
Heat lid	–	110	∞
Initial denaturation	1	98.0	120
Denaturation	30	98.0	10
Annealing	30	67.0	20
Extension	30	72.0	30
Final extension	1	72.0	420
Storage	–	4.0	∞

The amplified PCR products were purified using the GFX PCR DNA and Gel Band Purification Kit (Cytiva) according to the manufacturer's instructions.

Long ssDNAs were generated by Lambda Exonuclease digestion of the purified PCR-amplified dsDNA. The reaction components are listed in Table S9. The digestion was performed in a thermocycler using the parameters listed in Table S10. The digestion products were purified by agarose gel electrophoresis. The desired DNA bands were excised and recovered using the GFX PCR DNA and Gel Band Purification Kit (Cytiva).

Table S9: Components for Lambda Exonuclease digestion.

Components	V [µL]	c_{final}
dsDNA with one 5'-phosphate	20	26 ng/µL
Reaction buffer	5	1×
Lambda Exonuclease	1	0.2 U/µL
Water	24	–
Σ	50	–

Table S10: Lambda Exonuclease digestion parameters.

Reaction step	Cycle(s)	T [°C]	t [min]
Heat lid	–	80	∞
Lambda Exonuclease digestion	1	37	30
Deactivation	1	80	10
Storage	–	4	∞

2.1.4 Hybridization to the central hairpin-containing product

For targeted hybridization of the long ssDNAs to form the central hairpin-containing DNA product, 500 ng of each ssDNA strand were diluted in 30 µL of 1× TSE buffer. The solution was heated to 95 °C for 17 min and then allowed to hybridize by slow cooling to 4 °C at a rate of 1 °C every 12 min.

2.2 Ultrasound-induced scission of DNA

For sonication experiments, samples were prepared by diluting 1 µg of purified full-length DNA in 30 µL of 1× TSE buffer. The solution was then heated up to 85 °C for 5 min and subseqently hybridized by slowly cooling to 4 °C (1 °C/12 min).

Sonication for three technical replicates was carried out under ice cooling using a Q500 Sonicator (Qsonica, USA) fitted with a 3 mm diameter tip (422-A) and a single-tip horn. The operational frequency was 20 kHz and the amplitude was adjusted to 40% of its maximum (45 µm) and maintained consistently during the entire sonication process, independent of variations in concentration, temperature or viscosity of the samples. The energy input during sonication, labeled as E (in joules), was monitored to calculate the sonication power, represented as $P = Et^{-1}$ and the power intensity, also shown as $I_P = PA^{-1}$. t is considered as the effective sonication "on" time and A as the area of the probe tip. Pulsed sonication was used, alternating between 2.0 seconds "on" and 1.0 second "off". From this, the calculated power was $P = 2.92$ W and the power intensity was $I_P = 41.31$ W cm^{-2}. Subsequently, the products were concentrated (Eppendorf Concentrator plus) under high vacuum and 60 °C to be subsequently loaded in agarose gels for purification of product bands.

2.3 Capillary Electrophoresis

All measurements were performed on the Qsep1 system and were used for the kinetic analysis of Hp(0,6,4). Raw electropherogram data was recorded and subsequently plotted without prior smoothing or filtering. A High Resolution DNA Cartridge (BiOptic Inc.) was used for all experiments, with alignment markers at 20 bp and 1000 bp. Additional 80 bp dsDNA was added to the kinetic samples as an internal reference. Samples were injected at 8 kV for 10 s, and separation was carried out at 6 kV for 300 s. For data normalization, the electropherograms were aligned using the 20 bp and 1000 bp markers. The migration time of the 80 bp marker was defined as 0, while the migration time of the 1000 bp marker was defined as 1.

2.4 Illumina Sequencing

2.4.1 Experimental Procedure

For Illumina sequencing, all DNA resulting from ultrasound-induced scission was excised from the agarose gel after the reaction reached full conversion. For each experiment we chose three different technical replicates, *i.e.* separate mechanochemical reactions. For all three replicates, DNA was extracted from the agarose gel and subsequent library preparation was conducted using the NEBNext Ultra II DNA Library

Prep Kit. Notably, all three replicates were treated as independent, different samples for the library preparation. Afterwards, sequencing was performed with an Illumina MiSeq™ benchtop sequencer using a MiSeq Reagent Kit v2 Micro with 2×75 bp paired-end reads and 5 % PhiX (cat. no. FC-110-3001, Illumina).

2.4.2 Data Analysis

The obtained paired reads have been analyzed employing an in-house algorithm available on GitHub. Each 5'-end of a read is matched to a position within the known template sequence, being the sequence prior to any US-induced fragmentation, yielding a distribution of 5'-ends on each of the template strands. This algorithm works based on our previously published procedure,[1] but employs several critical differences:

- The algorithm decouples the paired reads and matches the single reads to either strand of the non-fragmented template sequence. This adaptation was done to go beyond fully paired double-stranded (*e.g.*, nicked) DNA sequences, in particular hairpin structures.
- Previously, we employed an algorithm that matches an initial segment of the read to the template and then allows for a defined number of mismatches for the remainder of the read. The improved implementation discards every single read that does not entirely match a subsequence on the template, increasing computational efficiency but losing more of the initial data.
- Breaking positions now are not necessarily integers. In cases of even sequence length, the zero position has been determined as the center between the two central bases, allowing fractional relative breaking positions such as $+1.5$. In case of odd sequence length, the sequence center is defined as the central base of the sequence; here the relative breaking positions are still integers.
- To account for heavy tailing of the observed breaking distributions, a Student's t-fit is employed (instead of a Gaussian fit) utilizing `scipy.stats` and `scipy.curve_fit`. Since the standard deviation σ is not always defined in this case, we use the interquartile range (IQR) as a measure of distribution width.

The histogram of 5'-ends (count of reads with a certain 5'-end) was normalized within a window of 50 bases of the sequence center, yielding breaking distributions. To validate the improved algorithm, we recalculated the original data from our previous publication, essentially recovering the qualitative findings from before (see Section 3.2.1). The ligation strategy leads to artifacts due to incomplete ligation, which are expressed as increased 5'-end counts at the ligation positions. To account for this, we replace the artificially increased breaking probability at the ligation sites by the average of the neighboring six base positions (three in each direction) and show the original fraction of reads as a gray bar. To validate that these artifacts are indeed due to the ligation and do not meaningfully influence the distribution parameters, we created a similar hairpin system through self-assembly (see Section 3.2.2). Additionally, we validate the Phred quality score and the fraction of reads that are discarded by the algorithm (see Section 3.2.4).

2.5 MD Simulations

The central sections of samples Hp(4,0,4), Hp(0,0,4), Hp(0,3,4), Hp(0,3,4)AT, Hp(0,4,4), Hp(0,4,4)AT, Hp(0,5,4)AT, Hp(0,5,4), Hp(0,6,4)AT, Hp(0,6,4), and Hp(0,10,4) have been covered in MD simulations. Starting from the center of the respective structure, 50 nucleotides in each direction have been selected on the non-hairpin-containing strand. The complementary bases from the hairpin strand and the secondary structure were also selected. In the case of Hp(4,0,4), we selected the central 52 bases in each direction from the center.

To obtain initial structures for these selected subsequences, a coarse-grained model of the structures was built *via* oxView[2] and then equilibrated using the in-built dynamics in oxView. The coarse-grained coordinates were then converted to an all-atom .pdb file using tacoxDNA.[3] The initial .pdb structures are provided on GitHub. Additionally, for each sample (expect the ones not experimentally covered) a partially broken system was created in order to mimic the first breakage on the non-hairpin (lower in the case of Hp(4,0,4)) strand. For this, in oxView, a nick was created between the central bases on the non-hairpin (lower in the case of Hp(4,0,4)) strand. We have previously shown[1] that the exact chemistry of the introduced termini does not significantly affect the resulting force profiles. Accordingly, chemically saturated alcohol and phosphate end groups were used in the present simulations, as generated

automatically by oxView. After this, the same procedure was applied to these samples. For systems only investigated through MD simulations Hp(0,3,4), Hp(0,3,4)AT, Hp(0,4,4)AT, Hp(0,5,4)AT, Hp(0,5,4), Hp(0,6,4)AT only the intact structure has been simulated corresponding to the first step of the proposed mechanism.

For the simulations, the OL21[4] force field was used in conjunction with the TIP3P water model. For all simulations, GROMACS 2025.2 was used.[5] The structures were solvated in a box with a size dependent on the number of bases contained in the hairpin strand (box dimensions between 10 nm $\times$ 10 nm $\times$ 87 nm and 15 $\times$ 15 nm $\times$ 102 nm). Sodium ions were added until a neutral box was reached. Each sample then underwent energy minimization. The resulting structure of the energy minimization underwent *NVT* and *NPT* equilibration, with each of them lasting 10 ns.

Constant-force simulations were conducted starting from the equilibrated structures using an applied force of 0.1 nN and 2.0 nN, respectively. The force is applied to each of the terminal nucleotides on both strands, being uniformly distributed on all atoms of the terminal residues, pulling the DNA in the $+z$ and $-z$ directions. These simulations were conducted for 100 ns each, with three (in cases Hp(0,3,4), Hp(0,3,4)AT, Hp(0,4,4)AT, and Hp(0,6,4)AT six) replicates per sample and force combination. The trajectory was written out every 10 ps, yielding 10,000 frames per simulation. The simulations of the same sequence share a common energy-minimized structure but undergo separate *NVT* and *NPT* equilibration. For sample Hp(0,6,4), additional simulations for the forces 0.5 nN, 1.0 nN, 1.5 nN, 2.5 nN, and 3.0 nN have been performed.

From the simulation data, we obtained the time-resolved distance between the terminal 5'-hydrogens as the end-to-end distance, which we display with a running average over 50 frames (0.5 ns), see Section 3.3.1. Additionally, the time-resolved distance for the backbone DNA bonds in all nucleotides is obtained. The first 20 ns of each simulation are discarded to obtain stable values after the external force has distributed within the molecule. For all simulations except the ones at 0.1 nN force, the bond distances are then averaged over the entire simulation run. The average distance $\overline{d}$ is then used to calculate the average force $\overline{F}$ utilizing a harmonic potential (see Equation 1).

$$\overline{F} = k \cdot (\overline{d} - d_0) \tag{1}$$

Here, k and d_0 are the spring constant and the equilibrium distance for the respective bond found in the OL21 force field. With this, we obtained force distributions along both strands of the samples on each of the backbone bonds (see Section 3.3.2).

The simulation runs at low force (0.1 nN) served as baseline simulations to correct for force-field intrinsic strains on certain bonds, even when no or small external force is applied. Again, the first 20 ns of each run were discarded. Then, an average bond distance was obtained by averaging the remaining frames across all three simulations. The average forces were calculated in the same procedure as above. Subtracting the average force on the baseline simulations from the average force of each run at higher forces, we obtained corrected force distributions (see Section 3.3.2). For sample Hp(0,6,4), we obtained a force calibration between 0.5 nN and 3.0 nN using this procedure (see Section 3.3.3).

3 Supporting Results

3.1 Capillary Electrophoresis

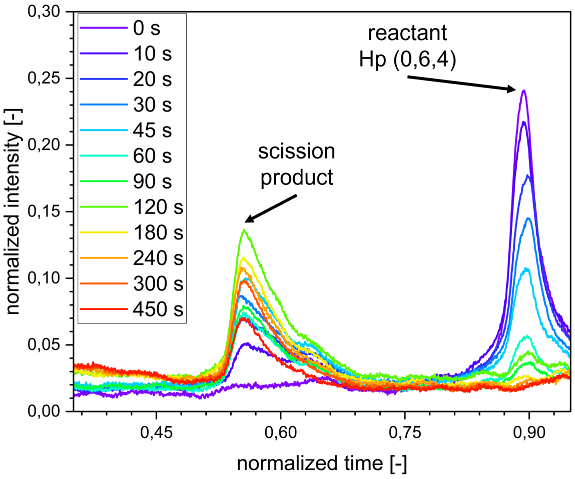

Figure S1: Normalized electropherograms of Hp(0,6,4) recorded up to sonication times of 7.5 min. Peaks corresponding to the reactant and the scission products are indicated.

3.2 NGS Analysis

3.2.1 Validation of Filtering Algorithm

Using the original data from our previous publication[1] employing the improved iteration of the algorithm, we obtain distribution centers μ and widths IQR displayed in Table S11. These values reproduce the qualitative findings from our previous study, verifying that the adaptations made to the algorithm capture the essentiality of the fragmentation within the NGS data. Additionally, we can make the direct comparison between the mechanophore-free nicked DNA systems and the biomechanophore systems employed in this study, showing that certain nicked samples of comparable size display a width up to almost twice that of the biomechanophore systems.

Table S11: Results for the nicked DNA samples from the previous publication evaluated from the exact same NGS datapoints using the improved algorithm and fitting scheme as discussed above. The major conclusions remain the same under the new algorithm, validating the approach. The errors are standard deviations.

Sample	Average relative μ	Average IQR
**AT***	-0.06 $\pm$ 0.09	10.03 $\pm$ 0.10
**GC***	1.94 $\pm$ 0.06	7.84 $\pm$ 0.20
**704***	0.74 $\pm$ 0.08	9.41 $\pm$ 0.75
**ATMi0***	0.26 $\pm$ 0.33	5.95 $\pm$ 0.52
**ATMi1***	0.31 $\pm$ 0.04	6.71 $\pm$ 0.36
**ATMi2***	0.39 $\pm$ 0.28	7.30 $\pm$ 0.08
**ATMi3***	-0.02 $\pm$ 0.53	7.98 $\pm$ 0.32
**ATMi4***	0.04 $\pm$ 0.15	7.41 $\pm$ 0.13
**400***	0.33 $\pm$ 0.15	9.48 $\pm$ 0.33
**1500***	-0.80 $\pm$ 0.37	12.94 $\pm$ 0.23

3.2.2 Effect of Ligation Strategy

To validate that the increased breaking probabilities at the ligation position are caused by the ligation, we used a control that is structurally identical to Hp(0,6,4) with a different nucleotide sequence but assembled by means of self-assembly, *i.e.*, PCR of two dsDNAs (with one 5-phosphorylated primer each) and subsequent λ-exonuclease digestion. The breaking distributions of this sample are shown in Figure S2, showing a similar sharp distribution on the non-hairpin strand and a wider distribution on the hairpin strand. The distribution parameters obtained by Student's t-fit are found as:

$$\mu_{\text{bot}} = -0.03 \pm 0.01 \quad \text{IQR}_{\text{bot}} = 3.82 \pm 0.72$$
$$\mu_{\text{top}} = 7.40 \pm 1.04 \quad \text{IQR}_{\text{top}} = 13.17 \pm 2.06$$

A statistical comparison using a one-way ANOVA test between the self-assembled samples and the similar Hp(0,6,4) samples obtained by ligation, where the ligation artifacts are replaced by the average of neighboring positions, yields no significant difference in distribution parameters of any strand ($p(\mu, \text{top}) = 0.887$, $p(\mu, \text{bot}) = 0.079$, $p(\text{IQR}, \text{top}) = 0.251$, $p(\text{IQR}, \text{bot}) = 0.076$). This validates that the ligation strategy combined with the modification made to the obtained distribution yields comparable distributions.

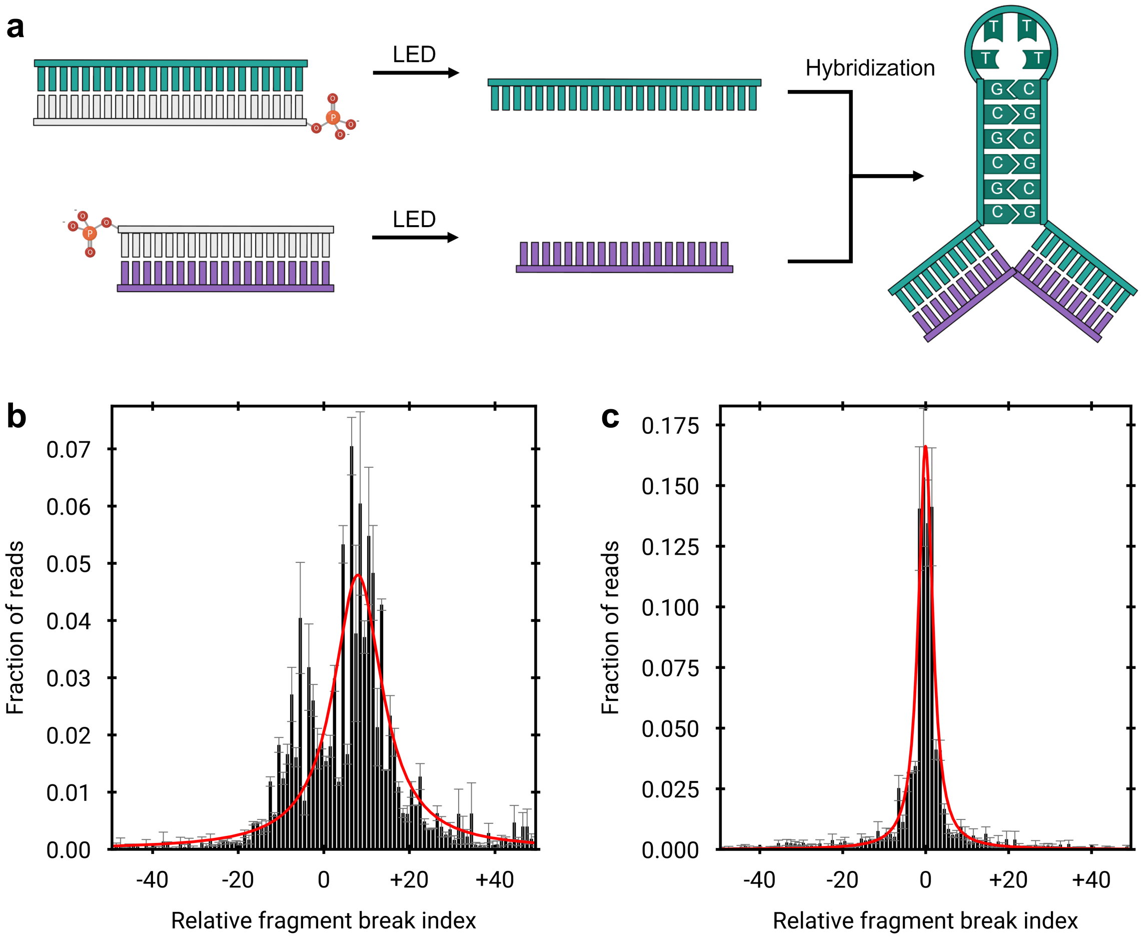

Figure S2: (a) Alternative synthesis route *via* λ-exonuclease (LED) and targeted hybridization to achieve $\sim$750 bp dsDNA with a central hairpin. Average breaking distributions for the **(b)** hairpin and **(c)** non-hairpin strand of the self-assembled Hp(0,6,4) system. Red lines denote Student's t distributions. Error bars are standard deviations ($n = 2$). The general shape of the distribution is conserved, but the artificially increased fraction of reads at the ligation positions is missing.

3.2.3 Comparison to dsDNA

As a control, ultrasonication of unmodified double-stranded DNA (dsDNA) was performed. The sequence of this dsDNA can be found in Fig. S24. Gel electrophoresis shows unspecific fragmentation ("smearing"),

as shown in Figure S3 a. Next-generation sequencing (NGS) likewise reports unspecific strand scission on both strands, with a slight bias toward the center of the sequence (Figure S3 b,c).

Notably, the maximum fraction of reads observed at any single position is approximately 0.8%. In comparison, the Hp(0,6,4) hairpin system exhibits approximately 12% of all breaks at a single position, corresponding to a 15-fold increase in localization.

Applying the normalization procedure described by Grokhovsky *et al.*,[6] we observe position-dependent increases and decreases in breaking probability in the range of 0.5–2.5 relative intensity, comparable in magnitude to the sequence-specific hotspots reported by by Grokhovsky *et al.* (see Figure S3 d,e). These effects are detectable in unmodified dsDNA but are negligible in hairpin-containing constructs, as hairpin-mediated scission occurs on a substantially higher scale.

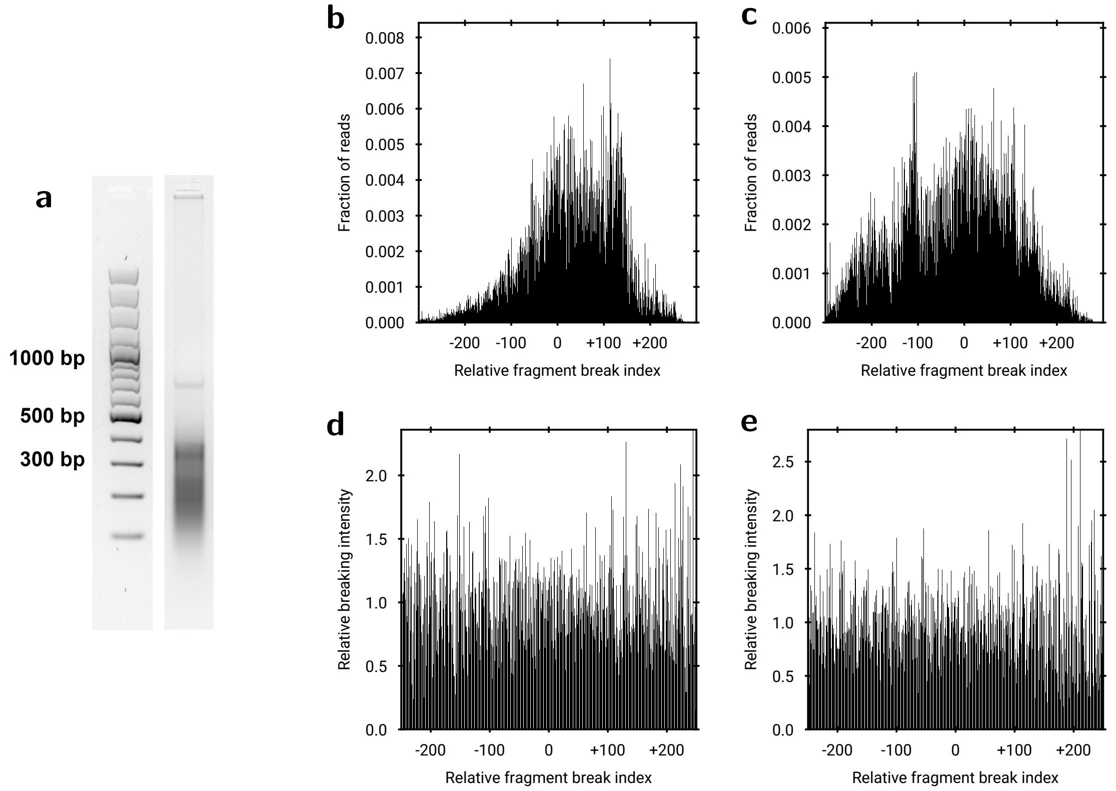

Figure S3: **(a)** Agarose gel electrophoresis of 706 bp long dsDNA after 7.5 min of sonication. Scission is unspecific as expected and no sharp bands are visible. **(b)** Average breaking distribution for the upper strand of the dsDNA under ultrasonication ($n = 2$). **(c)** Average breaking distributions for the non-hairpin strand of the dsDNA under ultrasonication ($n = 2$). **(d)** Relative breaking intensities on the upper strand of the dsDNA based on the upper strand breaking distribution using the window normalization (window size 15 bases in both directions) previously described.[6] **(e)** Relative breaking intensities on the non-hairpin strand of the dsDNA based on the non-hairpin strand breaking distribution using the window normalization (window size 15 bases in both directions) previously described.[6]

Additionally, we define an additional precision metric (denoted y) to quantify the fraction of breaks localized near the sequence center. This metric is calculated by dividing the sum of all reads within a window of ± 2 nt around the sequence center by the sum of all reads within a window of ± 300 nt. The larger window excludes terminal 5'-end reads, which are detected by NGS but do not correspond to strand scission events. For the lower strand in the case of unmodified dsDNA, the upper strand in case of nicked DNA (data from Ref. [1]), and for the non-hairpin strand in the Hp(0,6,4) system, this calculation was performed independently for each sequencing replicate, yielding:

$$y_{\text{dsDNA, lower}} = (1.36 \pm 0.04)\%$$
$$y_{\text{704*, upper}} = (19.2 \pm 1.1)\%$$
$$y_{\text{Hp(0,6,4), non-hairpin}} = (26.9 \pm 1.3)\%$$

These values show a very low central precision for dsDNA, much better precision for the previously described nicked system and more than 25% of all breaking events happening in a very narrow window for the hairpin system.

3.2.4 Filtering Accuracy and Read Quality

The Phred read quality, dependent on the base call position within a read, has been analyzed for all samples. This is shown exemplarily for Hp(0,6,4) in Figure S4 and for the remaining samples in Appendix Figure S27 and S28. Quality values show a high accuracy of the base call, even for the initial bases of each read.

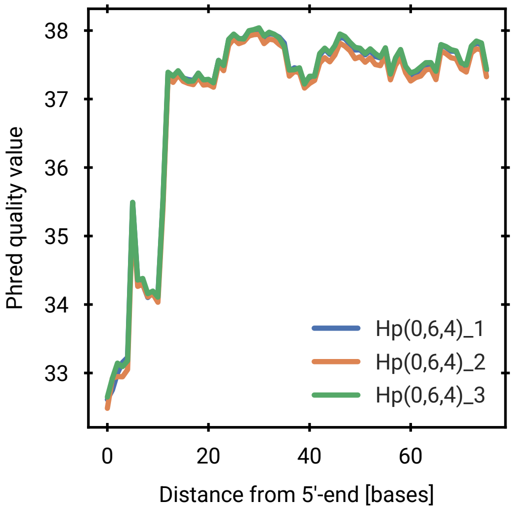

Figure S4: Phred quality values for sample Hp(0,6,4), showing a lower quality for the first bases of a read. The lowest quality value still yields an accuracy of $\gg 99.99$.

The filtering algorithm discards reads that cannot be matched to either strand of the template sequence. To quantify this, we provide the fraction of discarded reads in Table S12. These values are slightly increased compared to the previous implementation of the algorithm, but still capture a majority of the obtained reads with higher computational efficiency.

Table S12: Percentage of single reads that did not match for any of the two strands of the template structure.

Sample	Single read filtering accuracy [%]
Hp(4,0,4)_1	22.83
Hp(4,0,4)_2	18.61
Hp(4,0,4)_3	20.45
Hp(0,0,4)_1	16.83
Hp(0,0,4)_2	22.10
Hp(0,0,4)_3	23.22
Hp(0,4,4)_1	17.88
Hp(0,4,4)_2	14.85
Hp(0,4,4)_3	14.87
Hp(0,6,4)_1	16.76
Hp(0,6,4)_2	17.76
Hp(0,6,4)_3	17.73
Hp(0,10,4)_1	19.53
Hp(0,10,4)_2	17.93
Hp(0,10,4)_3	19.49
Hp(0,6,0)_1	15.97
Hp(0,6,0)_2	19.25
Hp(0,6,0)_3	18.36
Hp(0,6,8)_1	17.34
Hp(0,6,8)_2	20.65
Hp(0,6,8)_3	19.66
Hp(4,6,4)_1	19.63
Hp(4,6,4)_2	17.58
Hp(4,6,4)_3	16.09
Hp(5,6,4)_1	23.89
Hp(5,6,4)_2	17.00
Hp(5,6,4)_3	24.18
dsDNA_1	26.11
dsDNA_2	19.72

3.2.5 Breaking Distributions and Distribution Analysis

The average breaking distributions for both strands of the control sample Hp(4,0,4) are shown in Figure S5. We observe an overall very arbitrary breaking pattern with only a slight bias toward the center of the sequence. Individual breaking distributions of each replicate are shown in Appendix Figure S30 (lower strand) and Figure S29 (upper strand), respectively.

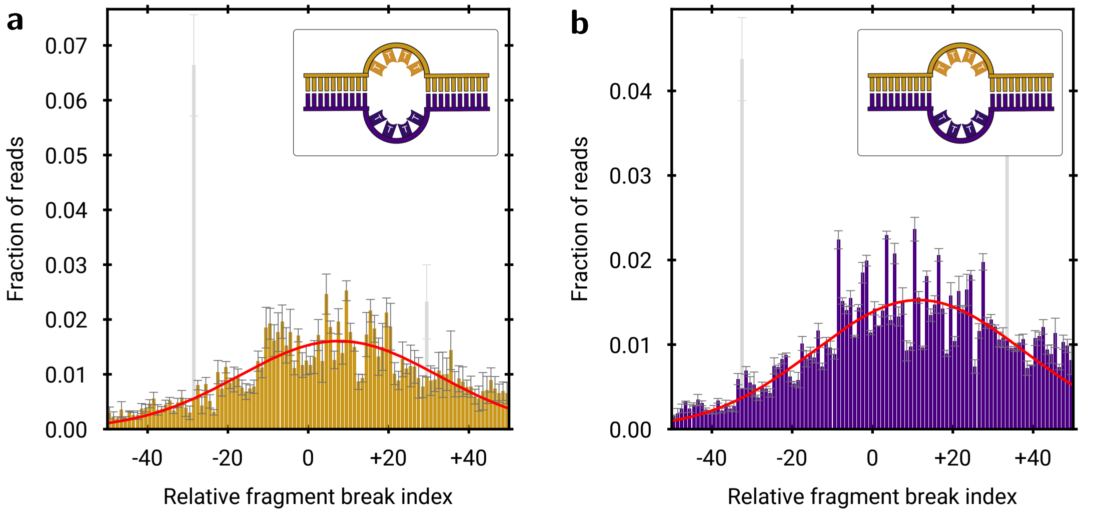

Figure S5: Average breaking distribution for the **(a)** upper and **(b)** lower strand of the control Hp(4,0,4) with no apparent mechanical activation. Red lines denote Student's t fit. Error bars are standard deviations ($n = 3$). Schematic structure of the Hp(4,0,4) sample is shown in the top right of the plots.

For the samples with varied stem size, the average distributions are shown in Figure S6. Individual breaking distributions for each of the replicates are shown in Appendix Figure S31 to S38. The remarkable difference on the non-hairpin strand between samples with a stem and Hp(0,0,4) has been discussed in the main text. For the hairpin strand, a pronounced shift in breaking distribution center with increasing stem size is observed, which might be caused by asymmetry in the sequence flanking the hairpin.

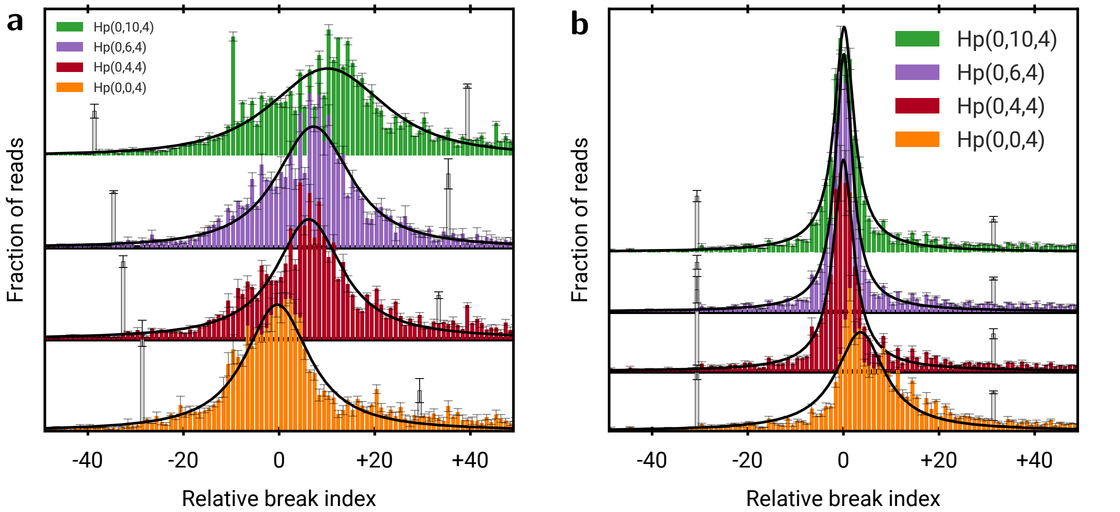

Figure S6: Average breaking distribution for the **(a)** hairpin and **(b)** non-hairpin strand of the stem variation samples (Hp(0,0,4), Hp(0,4,4), Hp(0,6,4), Hp(0,10,4)). Black lines denote Student's t fit. Error bars are standard deviations ($n = 3$).

For both strands, Student's t fits have been employed, yielding the IQR values displayed in Figure S7.

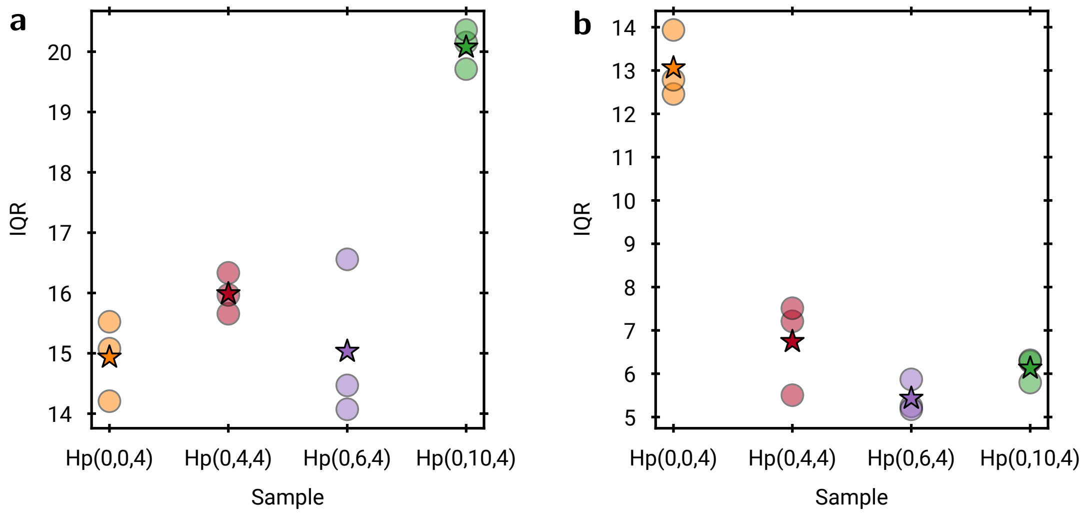

Figure S7: IQR values for the **(a)** hairpin and **(b)** non-hairpin strand of the stem variation samples. The star denotes the average. Hp(0,10,4) shows an increased distribution width on the hairpin strand, while Hp(0,0,4) shows an increased width on the non-hairpin strand.

Statistical analysis using a one-way ANOVA (values shown in Figures S8 and S9) shows that the IQRs on both strands are significantly different between the control Hp(4,0,4) and any of the stem variation samples. On the non-hairpin strand, the width obtained for Hp(0,0,4) is significantly different from any other sample; as discussed in the main text, this is due to increased concentration of the mechanical force in case a hairpin stem is present. On the hairpin strand, Hp(0,10,4) differs from the other samples, explained by a massively increased single-stranded domain within the second step of the breaking mechanism due to a larger hairpin stem being unfolded.

	HP4.0.4	HP0.0.4	HP0.4.4	HP0.6.4	HP0.10.4
HP4.0.4	1.000	**2.3e-04*** **	**2.6e-04*** **	**3.5e-04*** **	**7.7e-04*** **
HP0.0.4	**2.3e-04*** **	1.000	0.072	0.916	**2.8e-04*** **
HP0.4.4	**2.6e-04*** **	0.072	1.000	0.297	**1.2e-04*** **
HP0.6.4	**3.5e-04*** **	0.916	0.297	1.000	**0.003** **
HP0.10.4	**7.7e-04*** **	**2.8e-04*** **	**1.2e-04*** **	**0.003** **	1.000

Figure S8: p values of one-way ANOVA of Student's t fit parameter IQR for the hairpin strand distributions. The control sample Hp(4,0,4) has a significantly increased distribution width compared to any other sample. Additionally, Hp(0,10,4) has a significantly increased IQR compared to the other stem variation samples.

	HP4.0.4	HP0.0.4	HP0.4.4	HP0.6.4	HP0.10.4
HP4.0.4	1.000	**1.3e-06******	**1.6e-06******	**4.4e-08******	**2.6e-08******
HP0.0.4	**1.3e-06******	1.000	**0.001****	**1.1e-04*****	**1.3e-04*****
HP0.4.4	**1.6e-06******	**0.001****	1.000	0.119	0.396
HP0.6.4	**4.4e-08******	**1.1e-04*****	0.119	1.000	0.065
HP0.10.4	**2.6e-08******	**1.3e-04*****	0.396	0.065	1.000

Figure S9: p values of one-way ANOVA of Student's t fit parameter IQR for the non-hairpin strand distributions. The control sample Hp(4,0,4) has a significantly increased distribution width compared to any other sample. Additionally, Hp(0,0,4) has a significantly increased IQR compared to the other stem variation samples.

For samples with varying loop size (Hp(0,6,0), Hp(0,6,4) and Hp(0,6,8), see structures in Figure S10 a, we show the overlayed breaking distributions for both strands in Figure S10 c,d as well as the IQR values in Figure S10 b. The individual breaking distributions can be found in the Appendix Figures S35, S36 and S39 - S42. We find no significant difference in distribution width on the non-hairpin strand, indicating that the first step of the proposed mechanism is not depending on the loop length. This is reasonable since the tightening of the junction greatly depends on the stability of the stem, which is not varied by the loop modifications. Interestingly, we see differences on the hairpin-strand: For the largest loop size tested the distribution transforms into a bimodal distribution, which we have highlighted by employing a bimodal Student's t fit. This might be explained by the long sequence of thymines in the exposed single-stranded domain in the second step of the proposed mechanism, that might be less prone to break compared to the handles of the single-stranded domain.

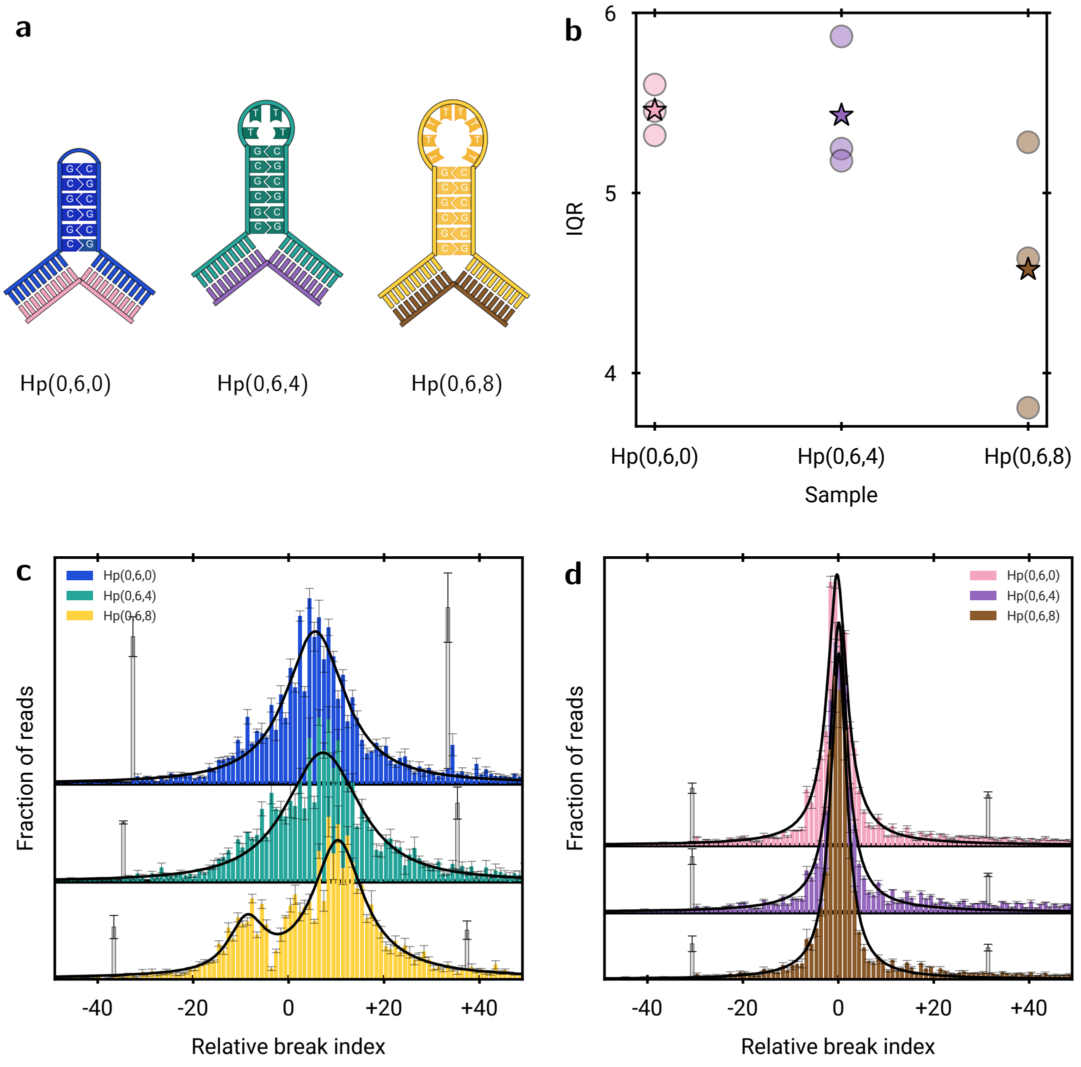

Figure S10: **(a)** Schematic structures of samples Hp(0,6,0), Hp(0,6,4) and Hp(0,6,8) varying the loop size. **(b)** IQR values of the Student's t distribution fits for the non-hairpin strand of the loop variation samples. **(c)** Overlayed breaking distributions for the hairpin strand of the loop variation samples. Black lines indicate Student's t fit or bimodal Student's t fit (for Hp(0,6,8). Error bars are standard deviations $(n = 3)$. **(d)** Overlayed breaking distributions for the non-hairpin strand of the loop variation samples. Black lines indicate Student's t fit. Error bars are standard deviations $(n = 3)$.

For samples with varying number of unpaired bases oposite of the hairpin (Hp(0,6,4), Hp(4,6,4) and Hp(5,6,4), see structures in Figure S11 a we show the overlayed breaking distributions for both strands in Figure S11 c,d as well as the IQR values in Figure S11 b. The individual breaking distributions can be found in the Appendix Figures S35, S36 and S43 - S46.

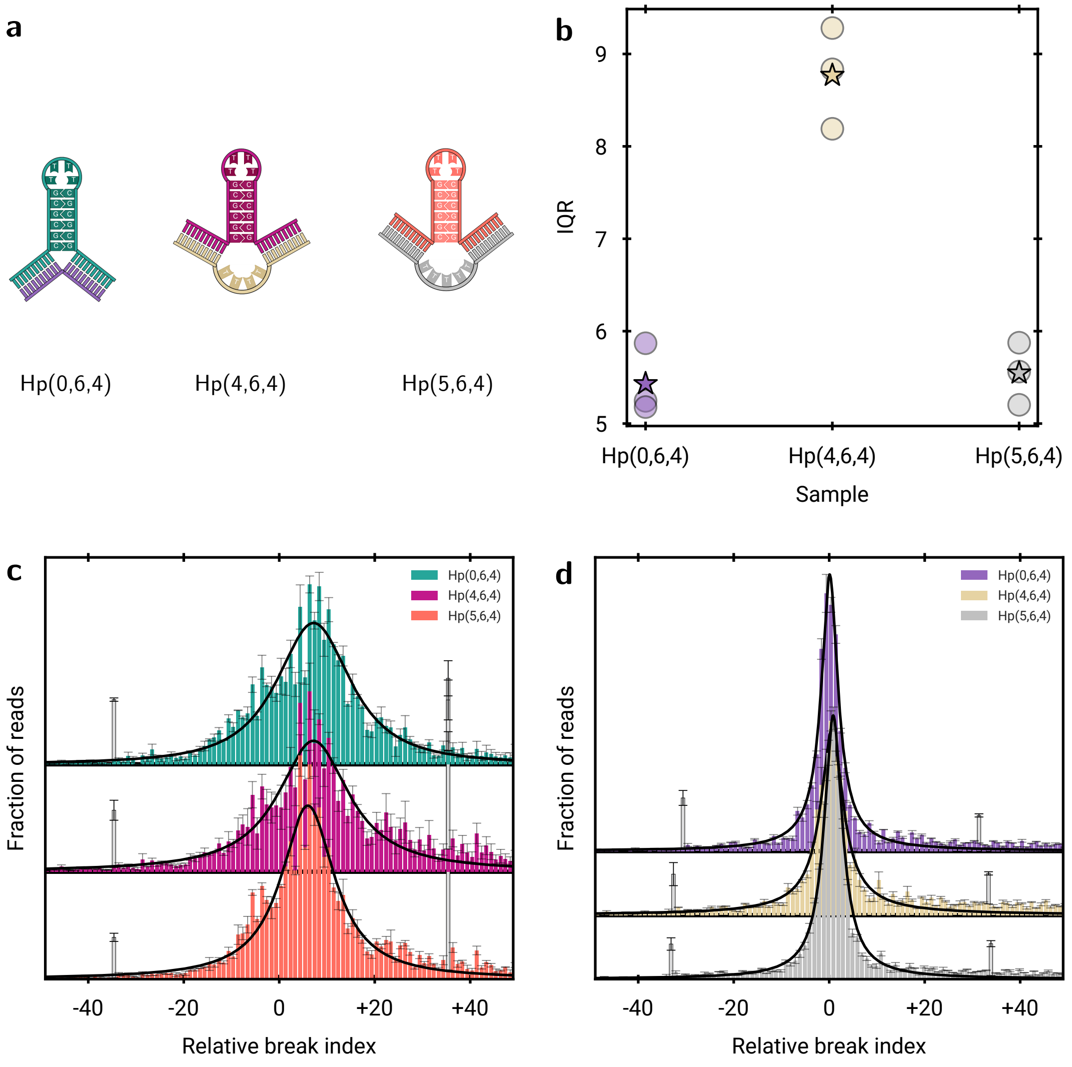

Figure S11: (a) Schematic structures of samples Hp(0,6,0), Hp(4,6,4) and Hp(5,6,4) varying the number of unpaired bases opposite of the hairpin. **(b)** IQR values of the Student's t distribution fits for the non-hairpin strand of the unpaired bases variation samples. **(c)** Overlayed breaking distributions for the hairpin strand of the unpaired bases variation samples. Black lines indicate Student's t fit. Error bars are standard deviations ($n = 3$). **(d)** Overlayed breaking distributions for the non-hairpin strand of the unpaired bases variation samples. Black lines indicate Student's t fit. Error bars are standard deviations ($n = 3$).

The variation of unpaired bases shows no difference on the breaking distribution of the hairpin strand. Interestingly, the non-hairpin strand shows a non-linear behavior: For zero and five unpaired bases the widht of the distribution does not differ significantly, but for four unpaired bases we observe a significant increase in distribution width. Notably, this increase is not as large as the increase observed when leaving out the hairpin stem. We hypnotize this being caused by changes in the torsional angle of the junction, that can significantly shift the force propagation profile.

3.3 MD Simulations

3.3.1 End-to-End Distances

The end-to-end distances for the samples Hp(0,0,4), Hp(0,4,4), Hp(0,6,4), and Hp(0,10,4) are shown in Figure S12 for external force 0.1 nN and in Figure S13 for external force 2.0 nN. For both forces, we see a larger end-to-end distance for the sample that is already broken opposite of the hairpin (prior to the second scission step) compared to the sample with intact structure (prior to the first scission step).

This difference is larger when a long hairpin sequence can be unfolded into a longer single-stranded domain. Additionally, we see the relaxation of the junction and unfolding of the hairpin as steps in the end-to-end distance for the samples with one broken strand. Naturally, these occur at different times for each simulation and happen simultaneously for simulations with 2.0 nN force.

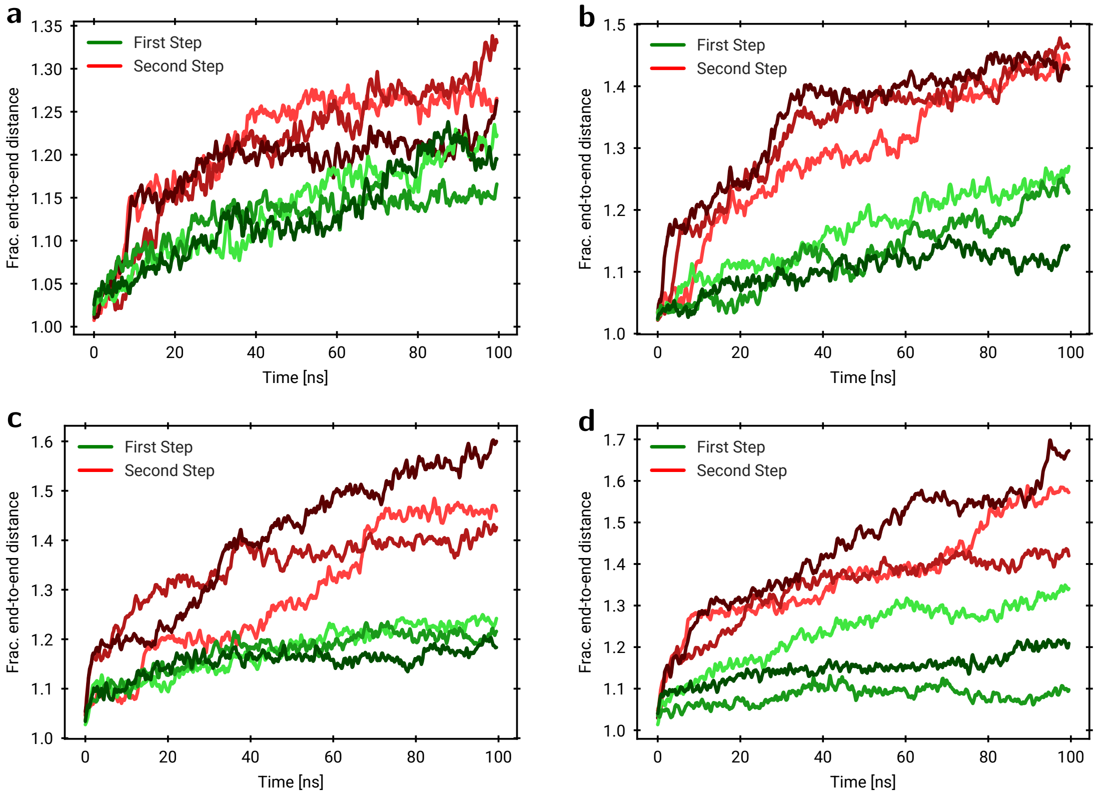

Figure S12: Running average of time-resolved end-to-end distances for prior to both scission steps for samples **(a)** Hp(0,0,4), **(b)** Hp(0,4,4), **(c)** Hp(0,6,4), and **(d)** Hp(0,10,4) for an external force of 0.1 nN.

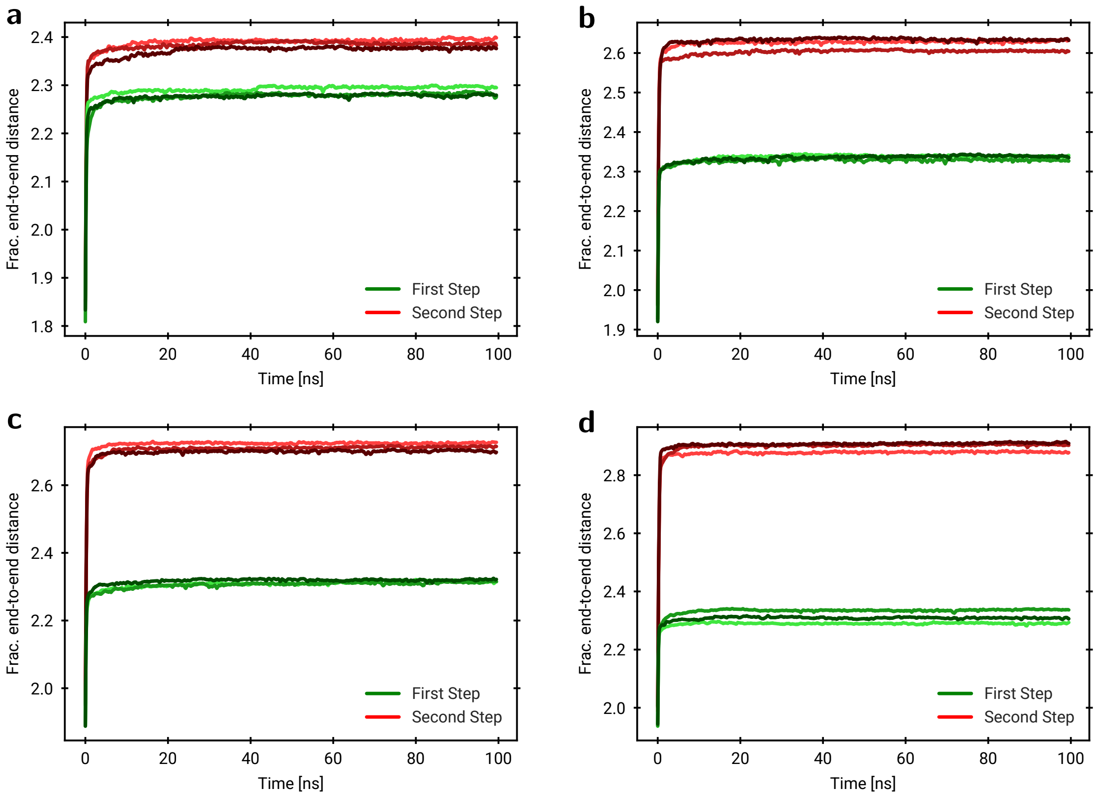

Figure S13: Running average of time-resolved end-to-end distances for prior to both scission steps for samples **(a)** Hp(0,0,4), **(b)** Hp(0,4,4), **(c)** Hp(0,6,4), and **(d)** Hp(0,10,4) for an external force of 2.0 nN.

3.3.2 Forces on Different Bond Types

The force distribution on each bond for the non-hairpin strand of sample Hp(0,6,4) at forces 0.1 nN and 2.0 nN is shown in Figure S14.

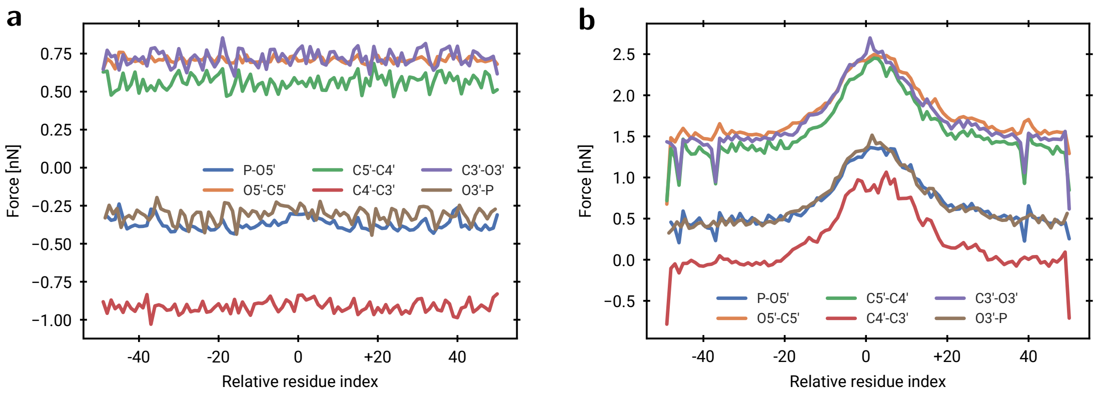

Figure S14: Force distribution along the non-hairpin strand of sample Hp(0,6,4) for each backbone bond type for **(a)** 0.1 nN and **(b)** 2.0 nN external force.

For the lower force, we observe no regional effect of the forces within the strand. For increased force, a clear increase toward the middle of the sequence is observed. For the remaining samples, these plots can be found in Appendix Figure S47 and S48. The different bond types show substantially different effective forces, even for the baseline sample, indicating that the force field intrinsically attributes higher forces to certain bond types, making a calibration by subtraction necessary. For Hp(0,6,4), the result of the calibration is shown in Figure S15; the remaining samples are shown in Appendix Figure S49.

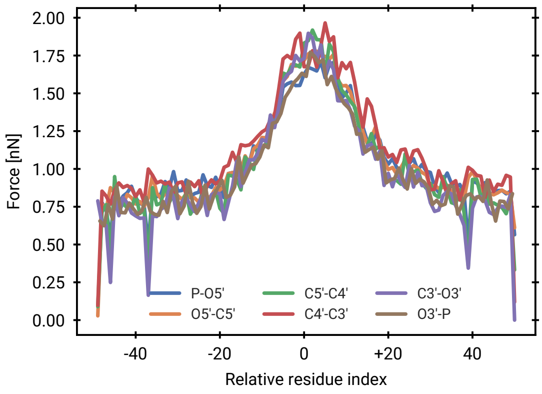

Figure S15: Corrected force distribution for the non-hairpin strand of sample Hp(0,6,4) at applied force of 2.0 nN subtracted by baseline value, showing no notable difference between different backbone bond types.

Here, we observe that all bond types show very similar force amplitudes, while the previous trend regarding the regional increase of forces is conserved. This indicates that only the dissociation energy of the respective bonds determines where the scission happens. Therefore, considering the literature, we select the C3'-O3' as the bond considered for the remainder of the structure, since it has been reported to be broken in the majority of ultrasonic DNA scission.[6,7]

3.3.3 Variation of Applied Forces

Forces in ultrasound have been reported to be in the nN range, even surpassing 3 nN.[6] To find the appropriate force for modeling our system in MD, we performed a force calibration in the range of 0.5 nN to 3.0 nN for the sample Hp(0,6,4). Force distributions at different external forces are shown in Figure S16. Here, we observe that, for forces below 2.0 nN, the signal-to-noise ratio is poor. Going beyond 2.0 nN, the force distribution widens, no longer discriminating between different positions along the structure. Therefore, we selected 2.0 nN as the force applied to the remaining systems.

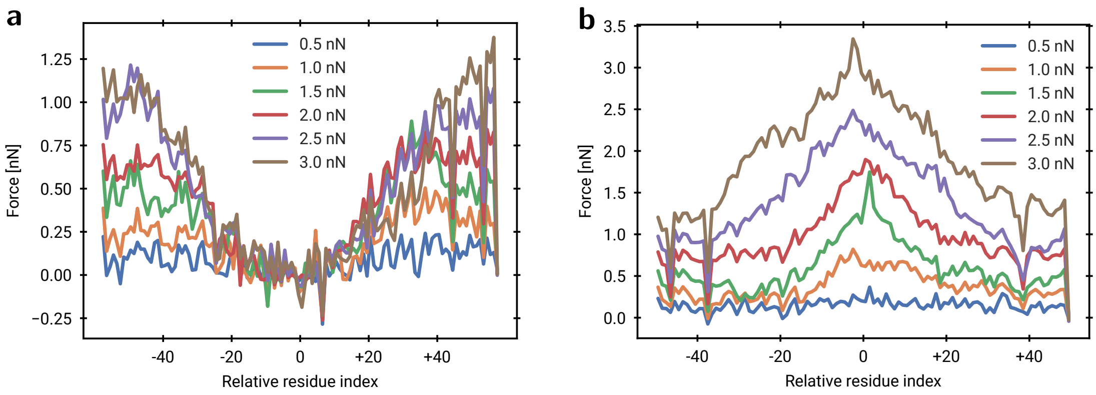

Figure S16: Corrected force distributions on the C3 -O3' bond of the **(a)** Hairpin and **(b)** non-hairpin strand of sample Hp(0,6,4) prior to the first scission at different external forces between 0.5 nN and 3.0 nN.

3.3.4 Additional Force Distributions

For completeness, we show the force distributions for the variation of stem length on both strands for both scission steps in Figure S17. For the first scission step, we observe a drastic decline of the effective force within the hairpin, while the forces are greatly increased on the opposing strand. For the second scission step, the picture is the opposite, since the non-hairpin strand is already broken, while the entire load is distributed to the now single-stranded domain. Here, the region of increased forces is much wider than in the previous step, explaining the wider breaking distribution observed in experiments.

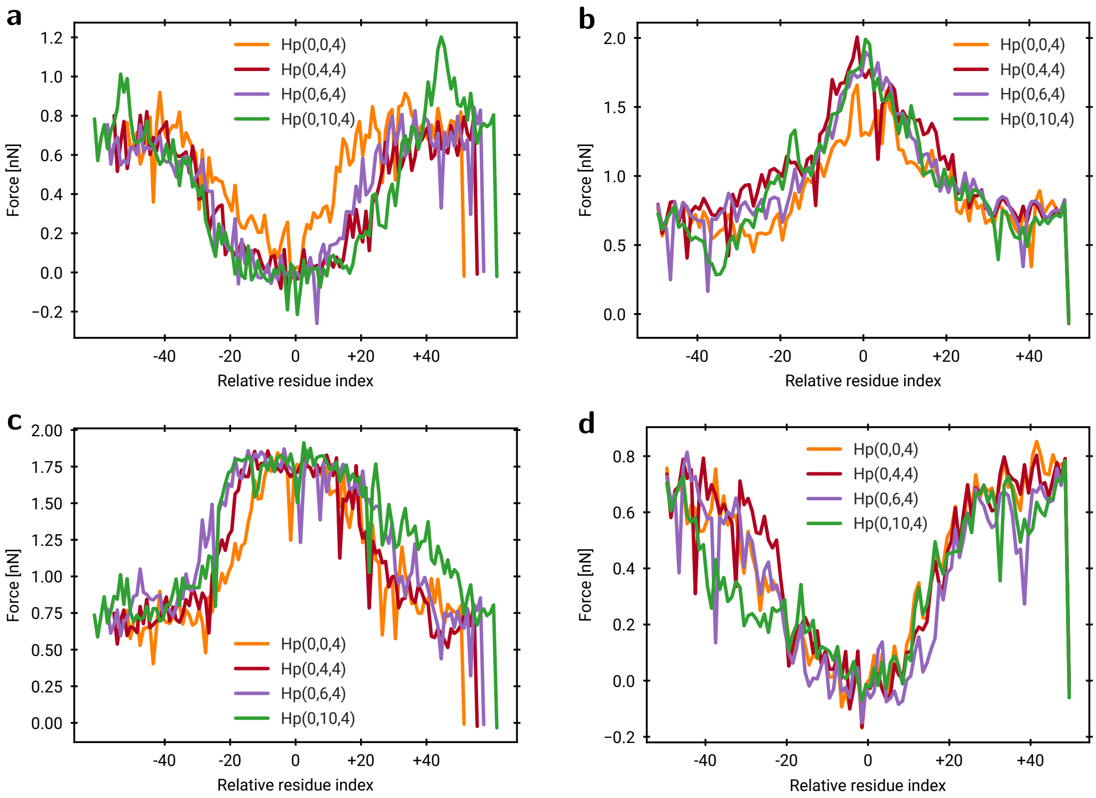

Figure S17: Corrected force distributions on the C3'-O3' bond for stem variation samples at external force of 2.0 nN shown for the **(a)** hairpin strand prior to first scission, **(b)** non-hairpin strand prior to first scission, **(c)** hairpin strand prior to second scission, and **(d)** non-hairpin strand prior to second scission.

3.3.5 Variation of the Base Composition of the Stem in MD Simulations

The samples Hp(0,3,4)AT, Hp(0,3,4), Hp(0,4,4)AT, Hp(0,4,4), Hp(0,5,4)AT, Hp(0,5,4), Hp(0,6,4)AT, and Hp(0,6,4) were investigated using molecular dynamics simulations to determine whether the base composition of the hairpin stem (AT vs. GC) affects the resulting force distribution along the non-hairpin strand.

As shown in Figure S18, no meaningful differences are observed between systems with identical stem lengths but different base compositions (*i.e.*, stems composed exclusively of AT base pairs compared to stems composed exclusively of GC base pairs). For each stem length, the force distributions along the non-hairpin strand remain essentially unchanged.

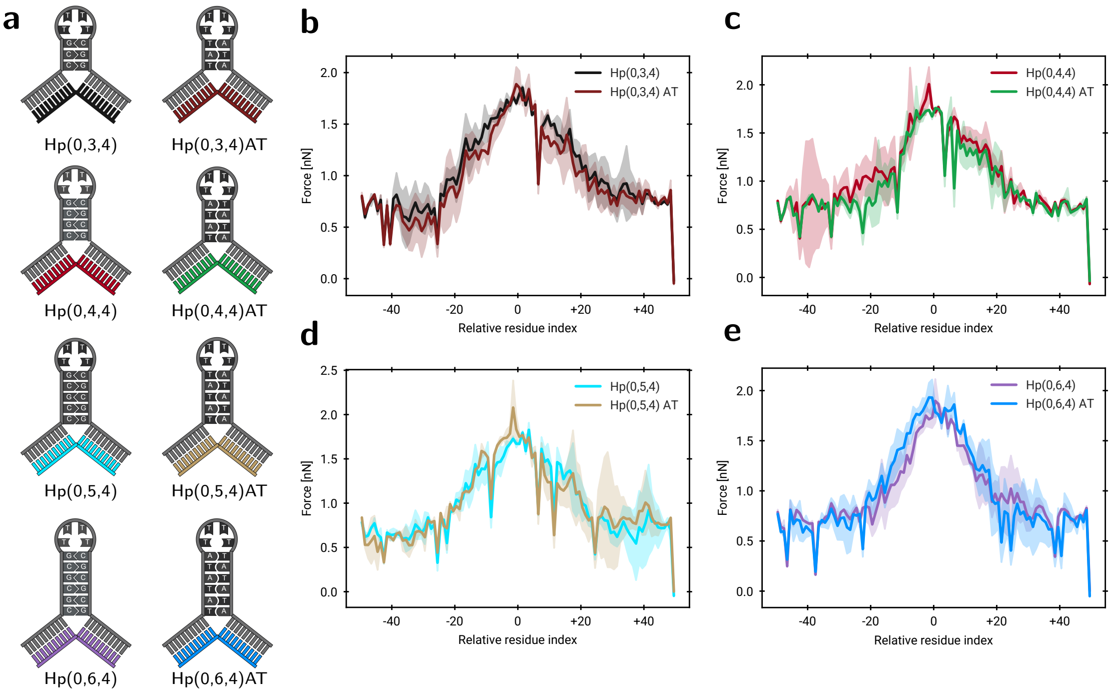

Figure S18: (a): Schematic structures of different hairpin systems with varied stem length and stem composition. Corrected force distributions on the C3'-O3' bond of the non-hairpin strand for stem variation samples at an external force of 2.0 nN, comparing all-GC and all-AT stem compositions at stem lengths of 3 bp **(b)**, 4 bp **(c)**, 5 bp **(d)**, and 6 bp **(e)**. Lines indicate the average across multiple independent MD simulation runs, and the colored areas denote the region spanned by $\pm$ s.d. $n = 3$ for Hp(0,4,4), Hp(0,5,4)AT, Hp(0,5,4), and Hp(0,6,4), and $n = 6$ for Hp(0,3,4), Hp(0,3,4)AT, Hp(0,4,4)AT, and Hp(0,6,4)AT.

In contrast, Figure S19 demonstrates that the stemless control system Hp(0,0,4) exhibits a markedly different force distribution compared to all structures containing a hairpin stem. Even relatively short stems consisting of only three base pairs produce a force distribution pattern characteristic of hairpin-containing structures, independent of whether the stem is composed of AT or GC base pairs.

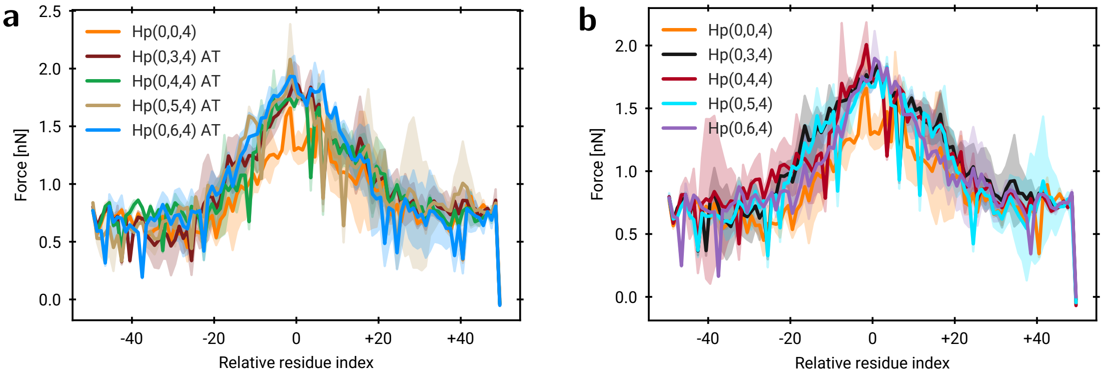

Figure S19: Corrected force distributions on the C3'-O3' bond of the non-hairpin strand for stem variation samples at an external force of 2.0 nN, comparing different stem lengths for stems that only contain AT base pairs **(a)** and stems that only contain GC base pairs **(b)**. Lines indicate the average across multiple independent MD simulation runs, and the colored areas denote the region spanned by $\pm$ s.d. $n = 3$ for Hp(0,0,4), Hp(0,4,4), Hp(0,5,4)AT, Hp(0,5,4), and Hp(0,6,4), and $n = 6$ for Hp(0,3,4), Hp(0,3,4)AT, Hp(0,4,4)AT, and Hp(0,6,4)AT.

These results indicate that the presence of a stable hairpin stem, rather than its base composition, is the primary determinant of the observed force distribution along the non-hairpin strand.

4 Appendix

4.1 DNA Sequences

```
  1 GTGCTCGTAG AACGTCCACG GACCTGCTTC TATTCGCGGA AGTGAATGGA TGCCACGTCG CGCATACGTC
 71 CAGTCTGGCT CCTGTTATCT TGGTACCTGG CCGGCTCGCC CGCGGTCGTG CCTTCTGTGC ATCGTCGGTA
141 ACCATAAGTC TTTAGTCCTC AAAGCCTCTG TAGCCGTTGG ATGGCGATCT TCCTGAGGTG ACATACGTCG
211 TCGTCCCCTC AAACTGGCAG ATGCACGGTT ACGATGCGCC CATCTACACC AACGTGGTTA CGAGAATTAC
281 GGTCAATCCG CCGTTTGTTC CCACGGAGAA TCCGACGGGT TGTTCTACGC CTCCATCCTT CTGGACCCAA
351 CTTAATCGCC TTGCAGCACA TCCCCCTTTA GCGAGCTGGC TTAATAGCGA AGAGGCCCGC ACCGATATGT
421 TCCGATAGTC CAGTCACCAC
```

Figure S20: Sequence **DNA**$_{\text{left}}$ for ligation route in $5' \rightarrow 3'$ direction. It contains the recognition site of BstXI (red), which, after restriction, exposes the sticky end CCTT (blue) which is needed for subsequent ligation to the middle part.

```
  1 GTGCTCGTAG AACGTCCACG GCCTGTTCCA CATCATAACG GCCATCCGAG CATGCACAGT CGCCGCACCT
 71 AAGACATAGC GCTCCTCTGT TCGTTAGTGT TCGCCGAGAC CATACGGTCA ATCCGCCGTT TAGATGAACT
141 TATTCACCGC TTCCTGATAG ATCTAATGTT CCTATGTGGT ACATCAAGTA TTACCTGATT CTCATGCGCT
211 ATATGGTAGC TTAAGACGAA ATAGATGAAT TATAGTTCAA CGCTGATGTC ACTTGGTCGT TGATGATTAG
281 TACATCTCTT TAATATTAAG ATAGCAGAAC CTGTAACATG TTCAGTTCTT TACGTCCGCT CATCAGCTCG
351 TAGGGCGAAG TGAAGAGAAT AGATAACTAA CTTGTGATGT GTTACATTTA ATCATCCAGC TTGTTTATGT
421 TCCGATAGTC CAGTCACCAC
```

Figure S21: Sequence **DNA**$_{\text{right}}$ for ligation route in $5' \rightarrow 3'$ direction. It contains the recognition site of Eco31I (red), which, after restriction, exposes the sticky end TCGC (blue) which is needed for subsequent ligation to the middle part.

```
  1 AATTCGAGCT CGGTACCCGG GGATCCTCTA GAGTCGACCT GCAGGCATGC AAGCTTGGCA CTGGCCGTCG
 71 TTTTACAACG TCGTGACTGG GAAAACCCTG GCGTTACCCA ACTTAATCGC CTTGCAGCAC ATCCCCCTTT
141 AGCGAGCTGG CTTAATAGCG AAGAGGCCCG CACCGATCGC CCTTCCCAAC AGTTGCGCAG CCTGAATGGC
211 GAATGGCGCT TTTCATGAAA AAGTCTTTAG TCCTCAAAGC CTCTGTAGCC GTTGGAGTGC GATCTTCCTG
281 AGGTGACATA CGTCGTCGTC CCCTCAAACT GGCAGATGCA CGGTTACGAT GCGCCCATCT ACACCAACGT
351 GTCGCGCGTT TTCGCGCGTG AAGAGCATTA CGGTCAATCC GCCGTTTGTT CCCACGGAGA ATCCGACGGG
421 TTGTTACTCG CTCACATTTA ATGTTGATGA AAGCTGGCTA CAGGAAGGCC AGACGCGAAT TATTTTTGAT
491 GGCGTTCCTA TTGGTTAAAA AATGAGCTGA TTTAACAAAA ATTTAATGCG AATTTTAACA AAATATTAAC
561 GTTTACAATT TAAATATTTG CTTATACAAT CTTCCTGTTT TTGGGGCTTT TCTGATTATC AACCGGGGTA
631 CATATGATTG ACATGCTAGT TTTACGATTA CCGTTCATCG ATTCTCTTGT TTGCTCCAGA CTCTCAGGCA
701 ATGACCTGAT AGCCTTTGTA
```

Figure S22: Sequence for hairpin strand of self-assembled dsDNA with a central hairpin *via* PCR and λ-exonuclease route in $5' \rightarrow 3'$ direction.

```
  1 TACAAAGGCT ATCAGGTCAT TGCCTGAGAG TCTGGAGCAA ACAAGAGAAT CGATGAACGG TAATCGTAAA
 71 ACTAGCATGT CAATCATATG TACCCCGGTT GATAATCAGA AAAGCCCCAA AAACAGGAAG ATTGTATAAG
141 CAAATATTTA AATTGTAAAC GTTAATATTT TGTTAAAATT CGCATTAAAT TTTTGTTAAA TCAGCTCATT
211 TTTTAACCAA TAGGAACGCC ATCAAAAATA ATTCGCGTCT GGCCTTCCTG TAGCCAGCTT TCATCAACAT
281 TAAATGTGAG CGAGTAACAA CCCGTCGGAT TCTCCGTGGG AACAAACGGC GGATTGACCG TAATGCTCTT
351 CAACACGTTG GTGTAGATGG GCGCATCGTA ACCGTGCATC TGCCAGTTTG AGGGGACGAC GACGTATGTC
421 ACCTCAGGAA GATCGCACTC CAACGGCTAC AGAGGCTTTG AGGACTAAAG ACTTTTTCAT GAAAAGCGCC
491 ATTCGCCATT CAGGCTGCGC AACTGTTGGG AAGGGCGATC GGTGCGGGCC TCTTCGCTAT TAAGCCAGCT
561 CGCTAAAGGG GGATGTGCTG CAAGGCGATT AAGTTGGGTA ACGCCAGGGT TTTCCCAGTC ACGACGTTGT
631 AAAACGACGG CCAGTGCCAA GCTTGCATGC CTGCAGGTCG ACTCTAGAGG ATCCCCGGGT ACCGAGCTCG
701 AATT
```

Figure S23: Sequence for non-hairpin strand of self-assembled dsDNA with a central hairpin *via* PCR and λ-exonuclease route in $5' \rightarrow 3'$ direction.

```
  1 TACAAAGGCT ATCAGGTCAT TGCCTGAGAG TCTGGAGCAA ACAAGAGAAT CGATGAACGG TAATCGTAAA
 71 ACTAGCATGT CAATCATATG TACCCCGGTT GATAATCAGA AAAGCCCCAA AAACAGGAAG ATTGTATAAG
141 CAAATATTTA AATTGTAAAC GTTAATATTT TGTTAAAATT CGCATTAAAT TTTTGTTAAA TCAGCTCATT
211 TTTTAACCAA TAGGAACGCC ATCAAAAATA ATTCGCGTCT GGCCTTCCTG TAGCCAGCTT TCATCAACAT
281 TAAATGTGAG CGAGTAACAA CCCGTCGGAT TCTCCGTGGG AACAAACGGC GGATTGACCG TAATGCTCTT
351 CATTACACGT TGGTGTAGAT GGGCGCATCG TAACCGTGCA TCTGCCAGTT TGAGGGGACG ACGACGTATG
421 TCACCTCAGG AAGATCGCAC TCCAACGGCT ACAGAGGCTT TGAGGACTAA AGACTTTTTC ATGAAAAGCG
491 CCATTCGCCA TTCAGGCTGC GCAACTGTTG GGAAGGGCGA TCGGTGCGGG CCTCTTCGCT ATTAAGCCAG
561 CTCGCTAAAG GGGGATGTGC TGCAAGGCGA TTAAGTTGGG TAACGCCAGG GTTTTCCCAG TCACGACGTT
631 GTAAAACGAC GGCCAGTGCC AAGCTTGCAT GCCTGCAGGT CGACTCTAGA GGATCCCCGG GTACCGAGCT
701 CGAATT
```

Figure S24: Sequence for unmodified, linear 706 bp dsDNA in $5' \rightarrow 3'$ direction.

```
1 GTGCT CGTAG AACGT CCACG G
1 GTGGT GACTG GACTA TCGGA ACAT
```

Figure S25: Primer sequences for ligation route in $5' \rightarrow 3'$ direction.

```
1 AATTC GAGCT CGGTA CCCGG
1 TACAA AGGCT ATCAG GTCAT TGCCT
```

Figure S26: Primer sequences for λ-exonuclease and PCR route in $5' \rightarrow 3'$ direction.

Table S13: DNA oligonucleotide strands used to assemble the central secondary motifs *via* the ligation route.

Strands	Sequence in $5' \rightarrow 3'$ direction
TopHp	ACGATGCGCCCATCTACACCAACGTGTCGCGCGTTTTCGCGC-<br>GTGAAGAGCATTACGGTCAATCCGCCGT
Top0S	ACGATGCGCCCATCTACACCAACGTGTTTTTTGAAGAGCATTA-<br>CGGTCAATCCGCCGT
Top4S	ACGATGCGCCCATCTACACCAACGTGTCGCGTTTTCGCGTGAA-<br>GAGCATTACGGTCAATCCGCCGT
Top10S	ACGATGCGCCCATCTACACCAACGTGTCGCGCGCGTTTTCG-<br>CGCGCGCGTGAAGAGCATTACGGTCAATCCGCCGT
Top0L	ACGATGCGCCCATCTACACCAACGTGTCGCGCGCGCGCGTGAA-<br>GAGCATTACGGTCAATCCGCCGT
Top8L	ACGATGCGCCCATCTACACCAACGTGTCGCGCGTTTTTTTTCGC-<br>GCGTGAAGAGCATTACGGTCAATCCGCCGT
BotHp0N	GCGAACGGCGGATTGACCGTAATGCTCTTCAACACGTTGGTGTA-<br>GATGGGCGCATCGTAAGG
BotHp4N	GCGAACGGCGGATTGACCGTAATGCTCTTCATTTTACACGTTGG-<br>TGTAGATGGGCGCATCGTAAGG
BotHp5N	GCGAACGGCGGATTGACCGTAATGCTCTTCATTTTTACACGTTG-<br>GTGTAGATGGGCGCATCGTAAGG

4.2 Sequencing Results

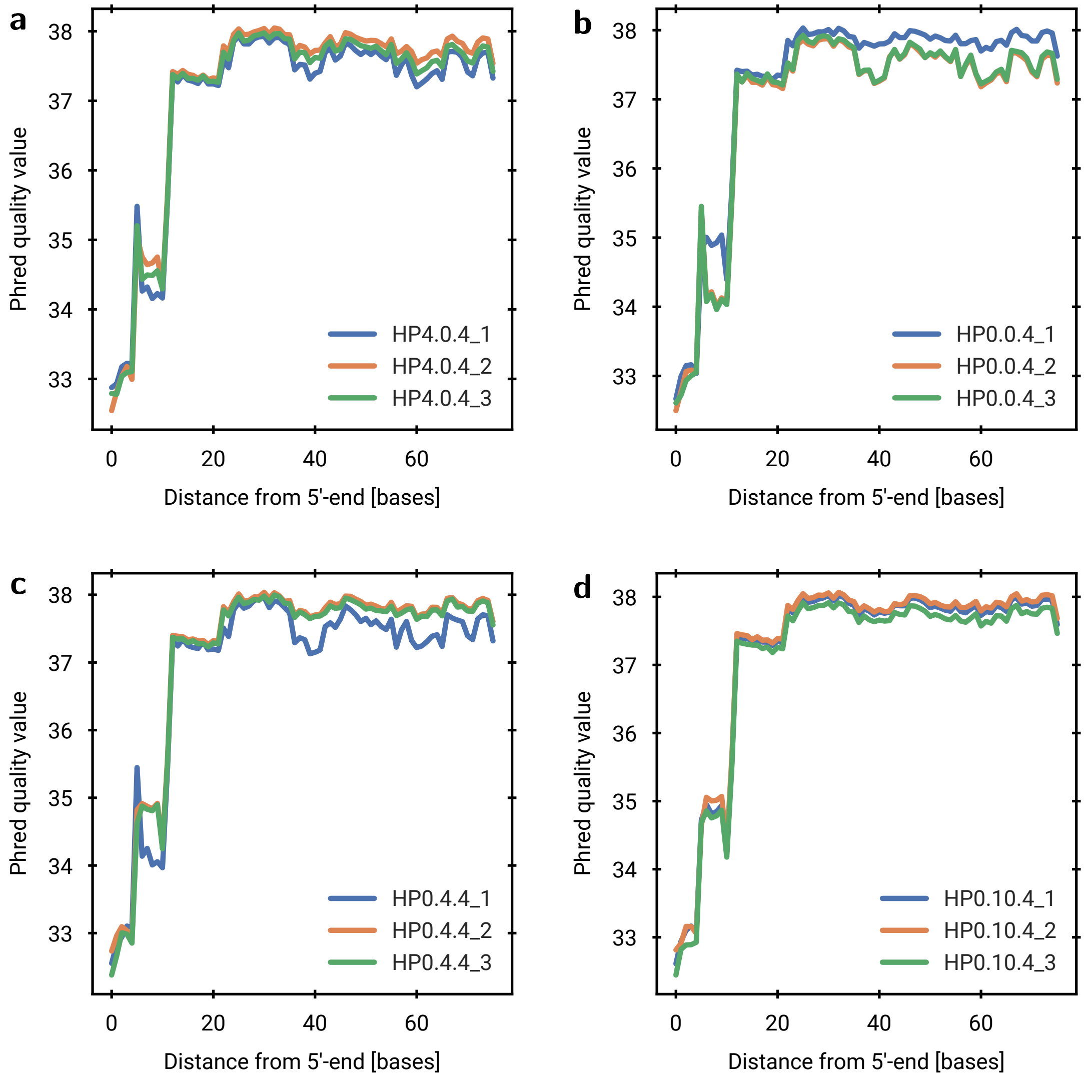

Figure S27: Phred quality values for samples **(a)** Hp(4,0,4), **(b)** Hp(0,0,4), **(c)** Hp(0,4,4), and **(d)** Hp(0,10,4) showing lower quality for the first bases of a read. The lowest quality value still yields an accuracy of $\gg 99.99$.

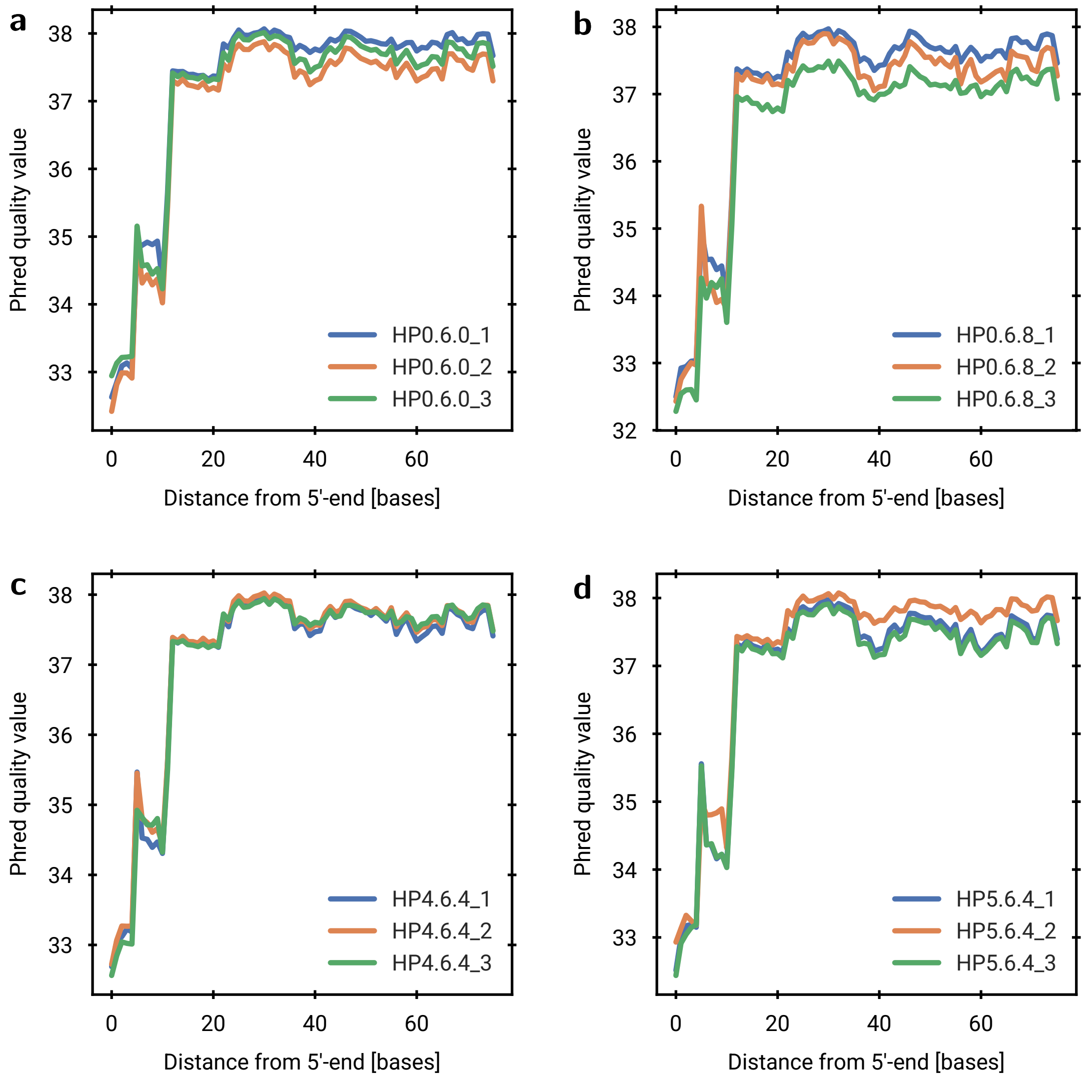

Figure S28: Phred quality values for samples **(a)** Hp(0,6,0), **(b)** Hp(0,6,8), **(c)** Hp(4,6,4), and **(d)** Hp(5,6,4) showing lower quality for the first bases of a read. The lowest quality value still yields an accuracy of $\gg 99.99$.

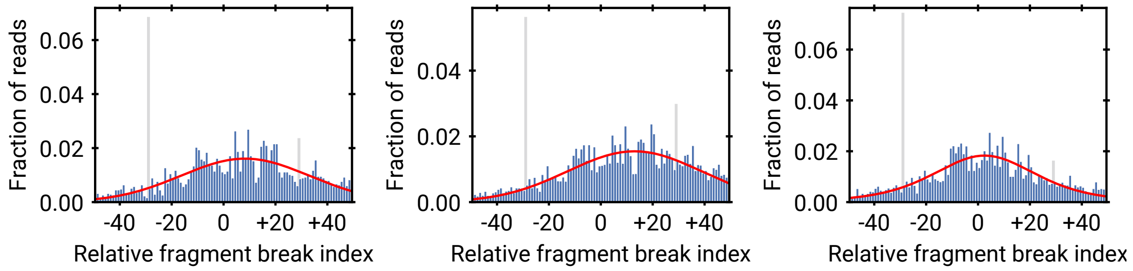

Figure S29: Individual breaking distributions for the upper strand of the three replicates of sample Hp(4,0,4). Red lines denote the Student's t fit.

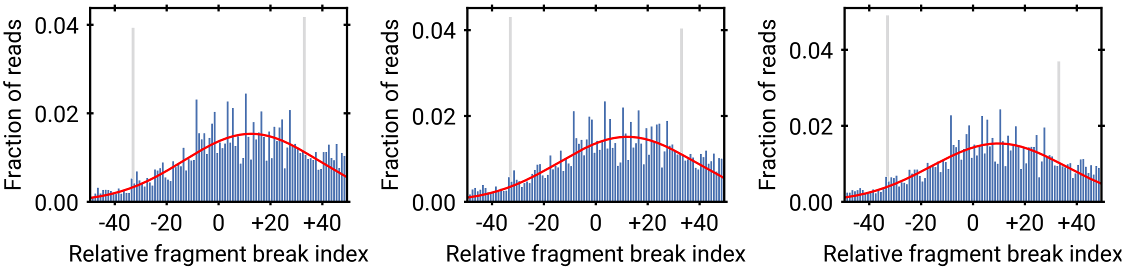

Figure S30: Individual breaking distributions for the lower strand of the three replicates of sample Hp(4,0,4). Red lines denote the Student's t fit.

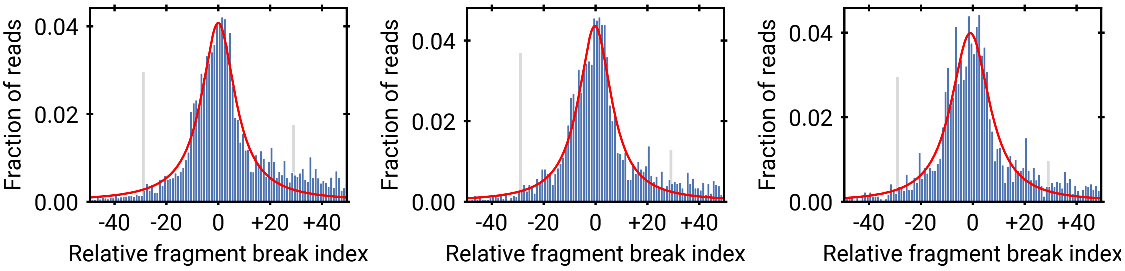

Figure S31: Individual breaking distributions for the hairpin strand of the three replicates of sample Hp(0,0,4). Red lines denote the Student's t fit.

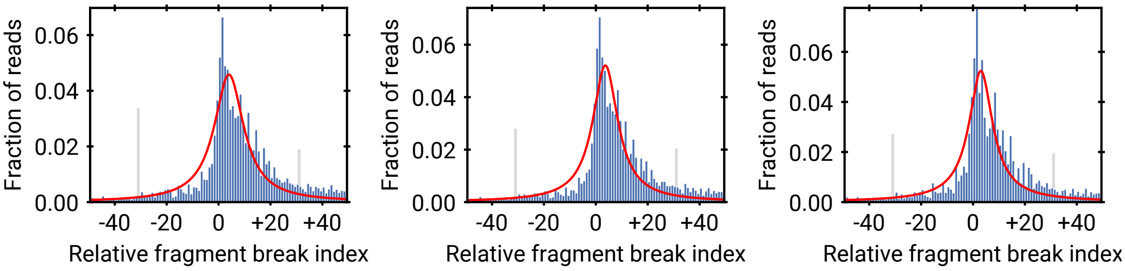

Figure S32: Individual breaking distributions for the non-hairpin strand of the three replicates of sample Hp(0,0,4). Red lines denote the Student's t fit.

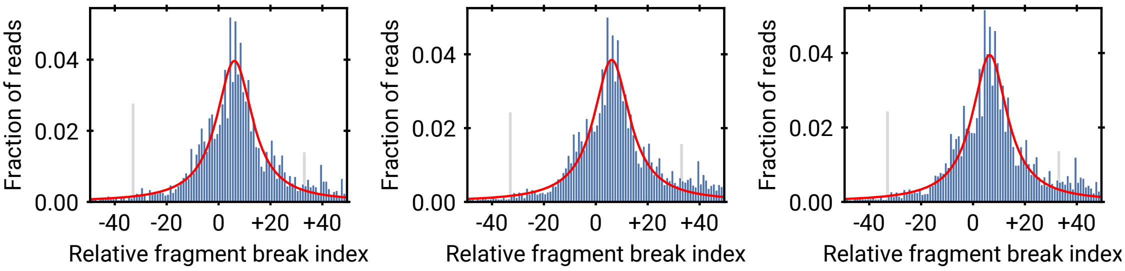

Figure S33: Individual breaking distributions for the hairpin strand of the three replicates of sample Hp(0,4,4). Red lines denote the Student's t fit.

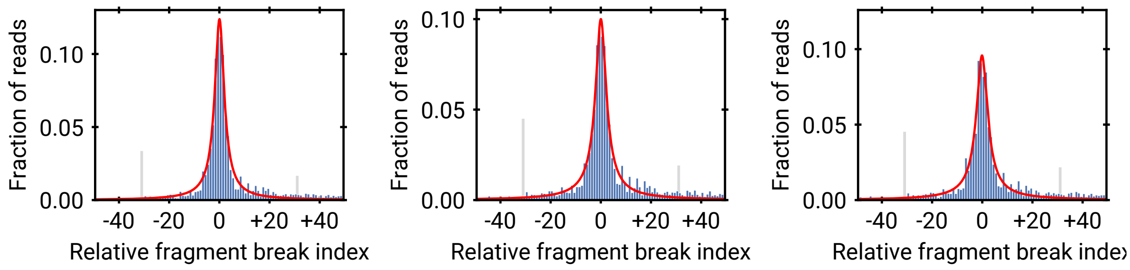

Figure S34: Individual breaking distributions for the non-hairpin strand of the three replicates of sample Hp(0,4,4). Red lines denote the Student's t fit.

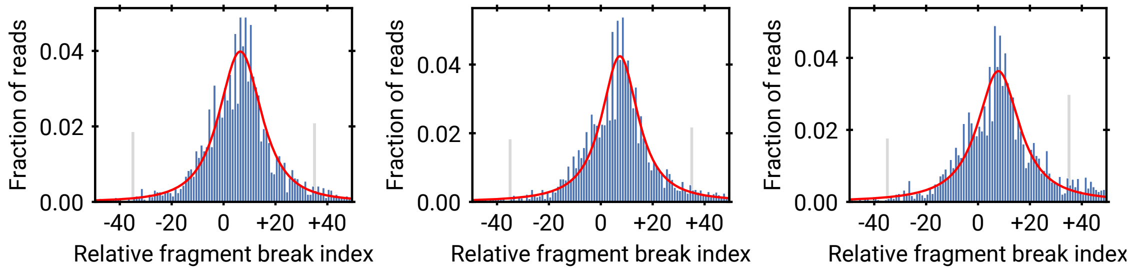

Figure S35: Individual breaking distributions for the hairpin strand of the three replicates of sample Hp(0,6,4). Red lines denote the Student's t fit.

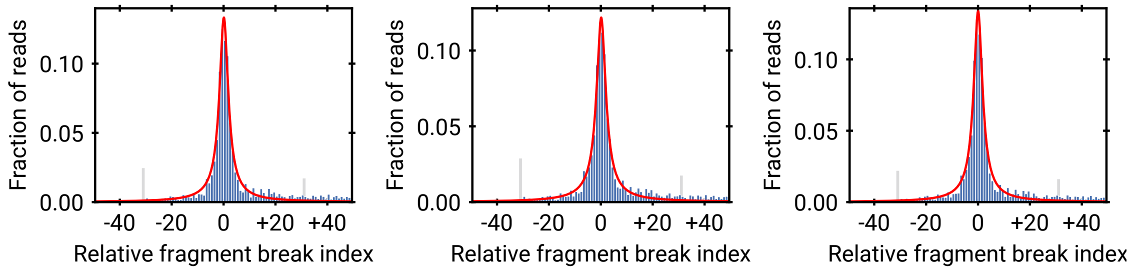

Figure S36: Individual breaking distributions for the non-hairpin strand of the three replicates of sample Hp(0,6,4). Red lines denote the Student's t fit.

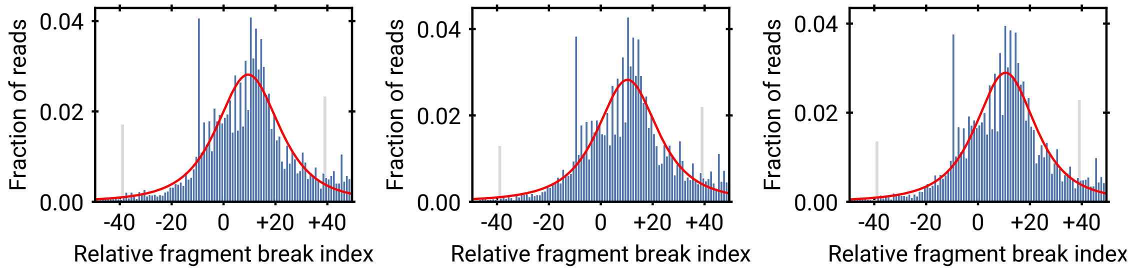

Figure S37: Individual breaking distributions for the hairpin strand of the three replicates of sample Hp(0,10,4). Red lines denote the Student's t fit.

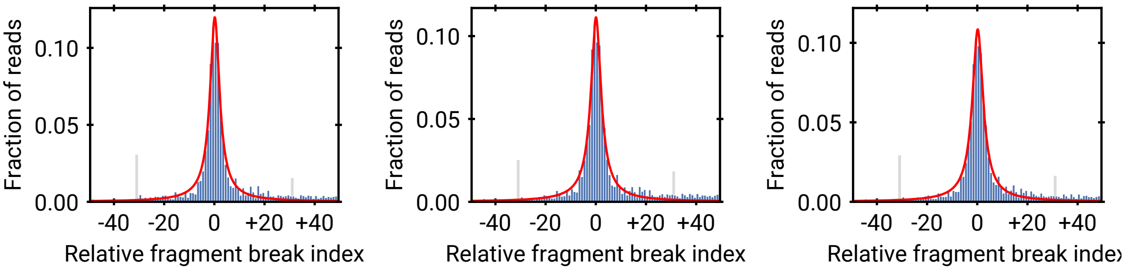

Figure S38: Individual breaking distributions for the non-hairpin strand of the three replicates of sample Hp(0,10,4). Red lines denote the Student's t fit.

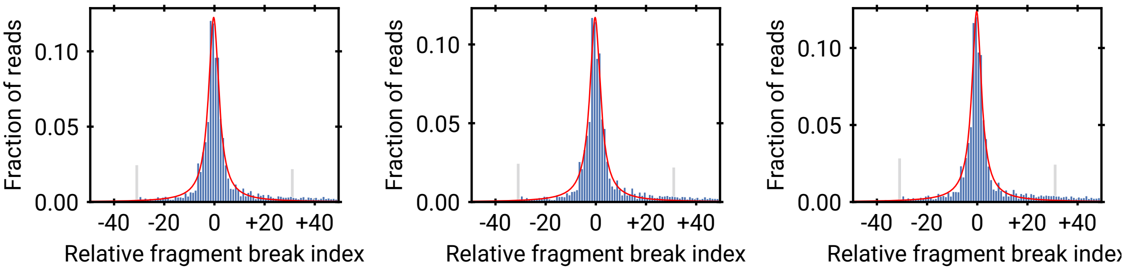

Figure S39: Individual breaking distributions for the non-hairpin strand of the three replicates of sample Hp(0,6,0). Red lines denote the Student's t fit.

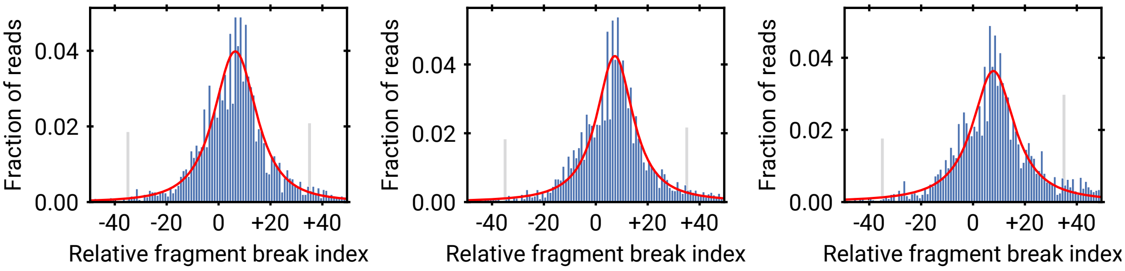

Figure S40: Individual breaking distributions for the hairpin strand of the three replicates of sample Hp(0,6,0). Red lines denote the Student's t fit.

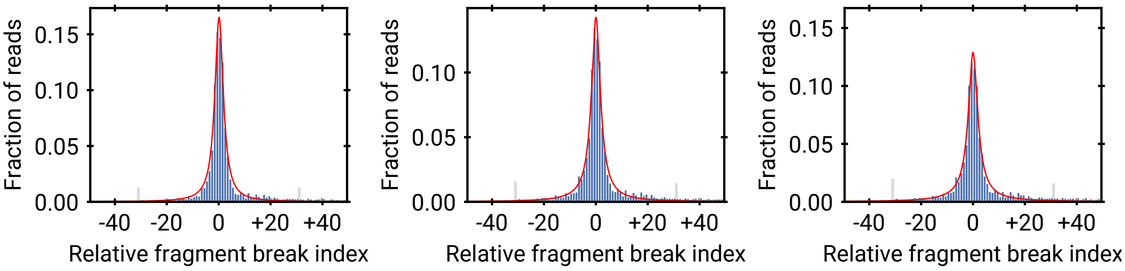

Figure S41: Individual breaking distributions for the non-hairpin strand of the three replicates of sample Hp(0,6,8). Red lines denote the Student's t fit.

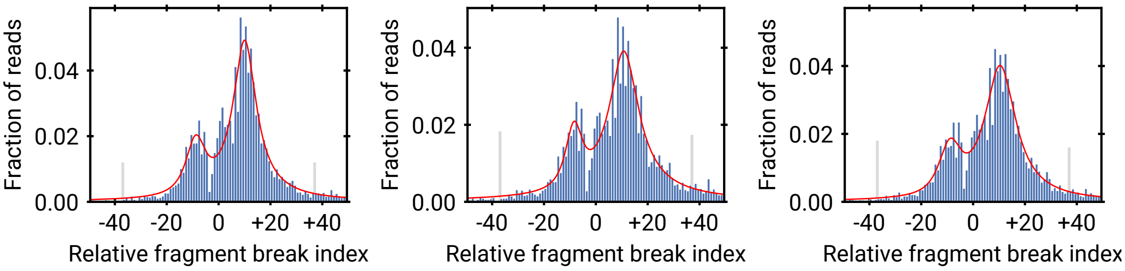

Figure S42: Individual breaking distributions for the hairpin strand of the three replicates of sample Hp(0,6,8). Red lines denote a bimodal Student's t fit.

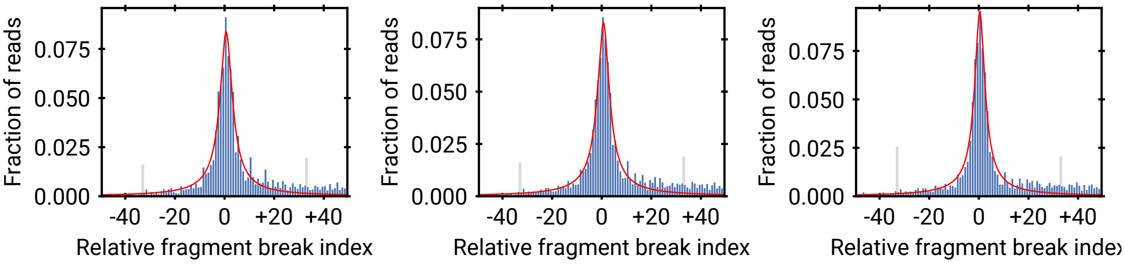

Figure S43: Individual breaking distributions for the non-hairpin strand of the three replicates of sample Hp(4,6,4). Red lines denote the Student's t fit.

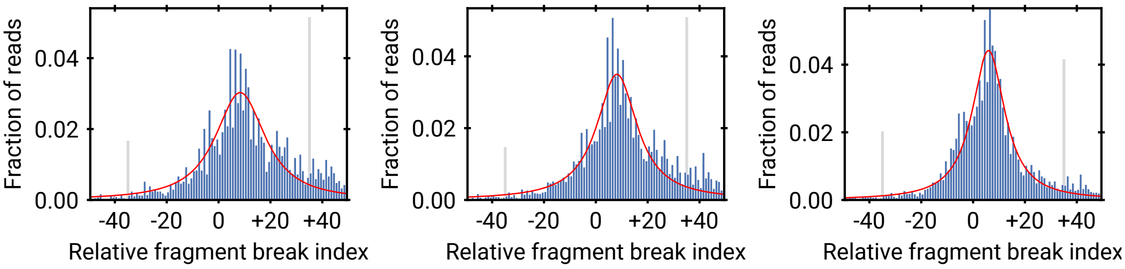

Figure S44: Individual breaking distributions for the hairpin strand of the three replicates of sample Hp(4,6,4). Red lines denote the Student's t fit.

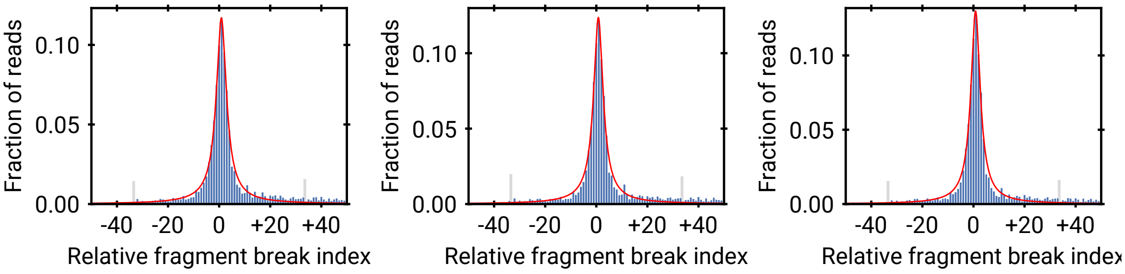

Figure S45: Individual breaking distributions for the non-hairpin strand of the three replicates of sample Hp(5,6,4). Red lines denote the Student's t fit.

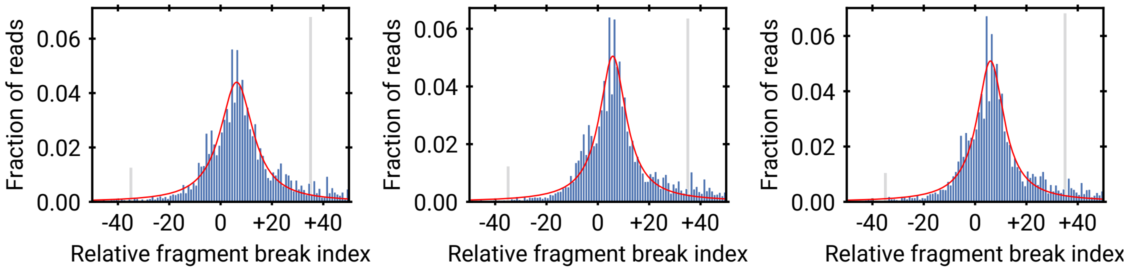

Figure S46: Individual breaking distributions for the hairpin strand of the three replicates of sample Hp(5,6,4). Red lines denote the Student's t fit.

4.3 MD Simulations

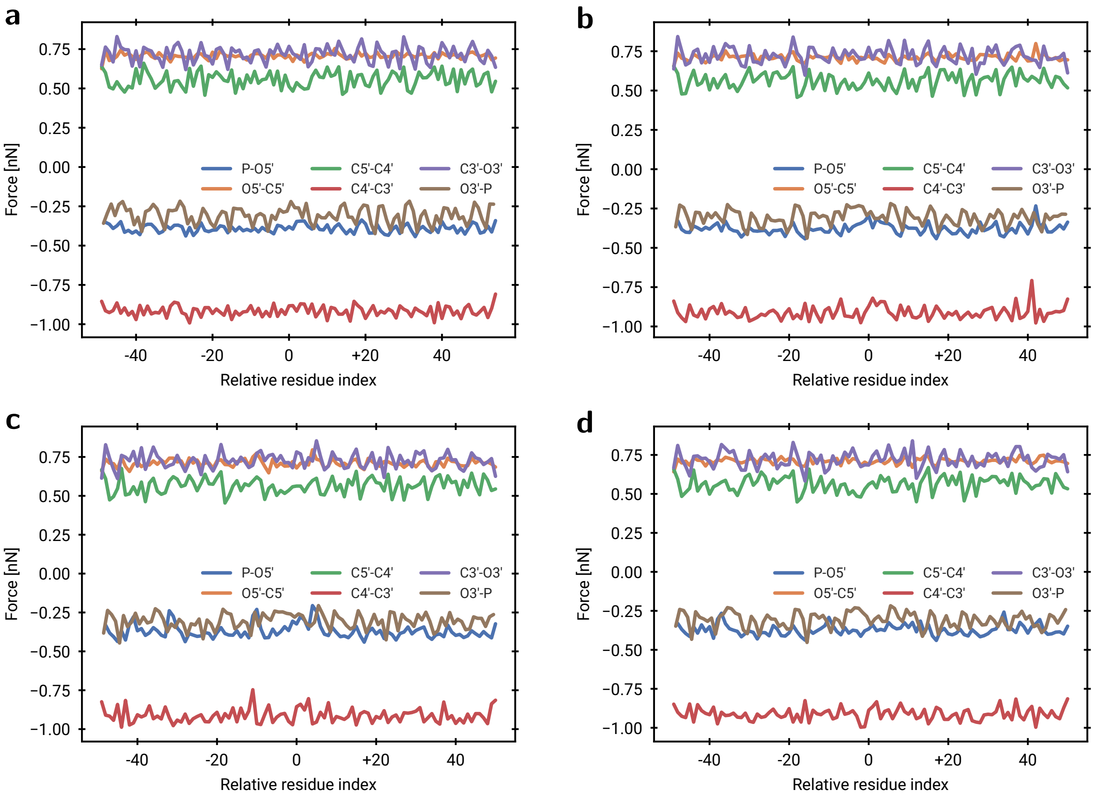

Figure S47: Force distribution on the non-hairpin strand of sample **(a)** Hp(4,0,4), **(b)** Hp(0,0,4), **(c)** Hp(0,4,4), and **(d)** Hp(0,10,4) for different backbone bonds at external force 0.1 nN.

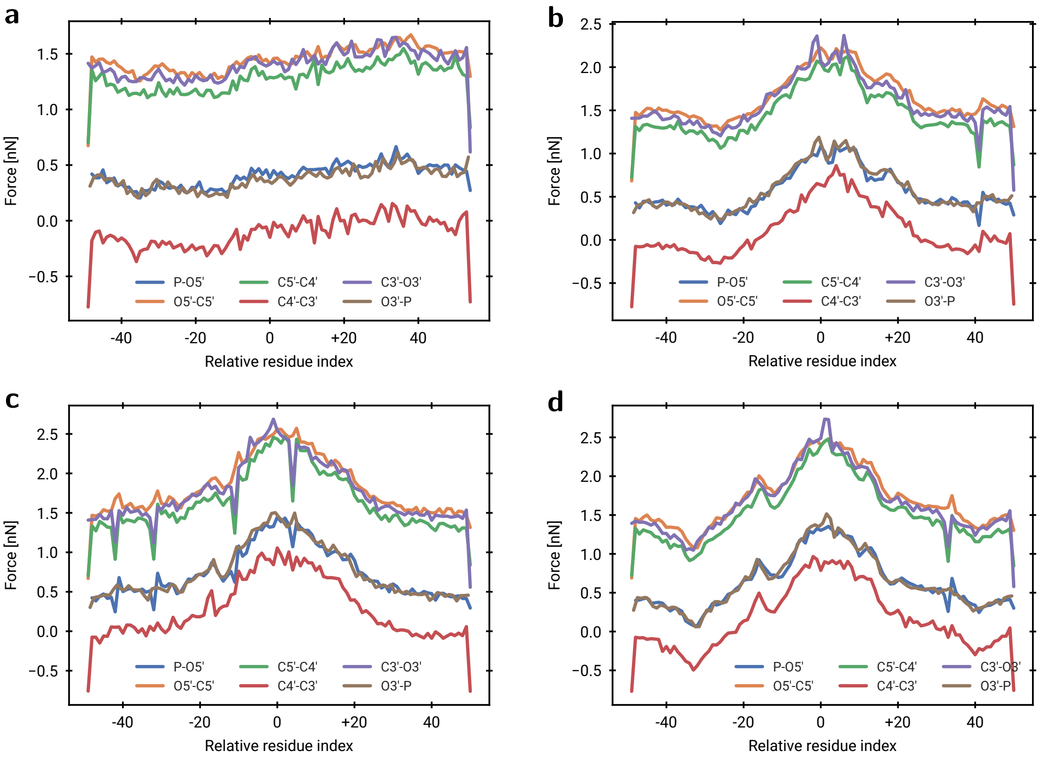

Figure S48: Force distribution on the non-hairpin strand of sample **(a)** Hp(4,0,4), **(b)** Hp(0,0,4), **(c)** Hp(0,4,4), and **(d)** Hp(0,10,4) for different backbone bonds at external force 2.0 nN.

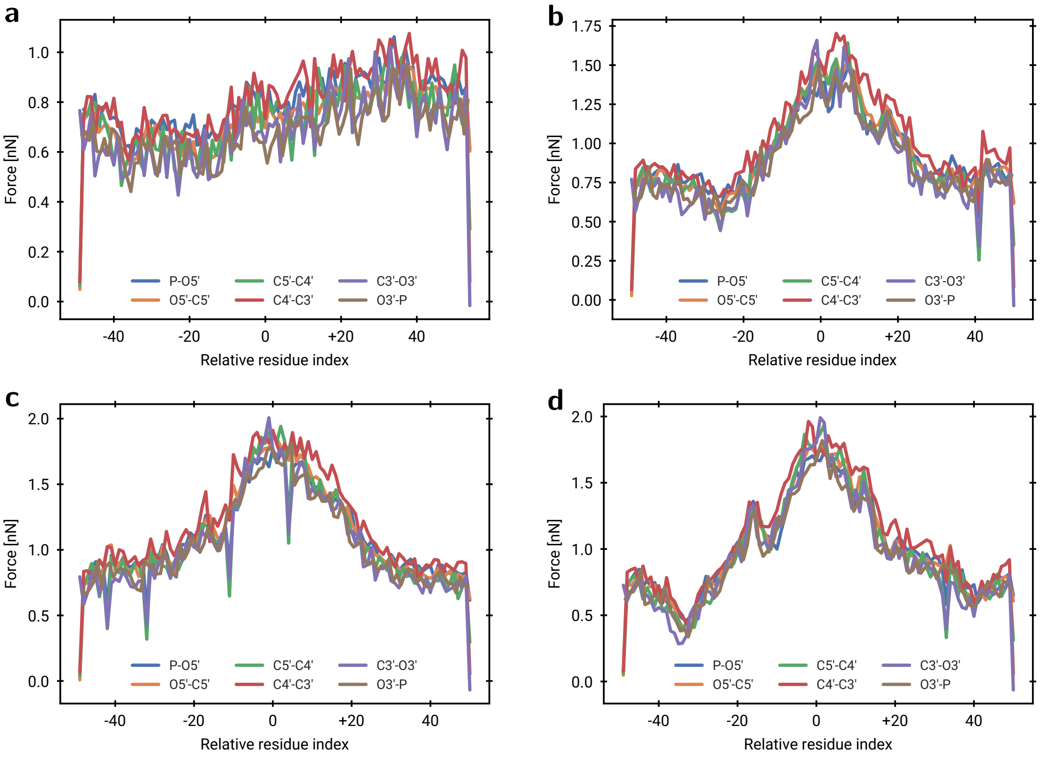

Figure S49: Corrected force distribution for the non-hairpin strand of sample **(a)** Hp(4,0,4), **(b)** Hp(0,0,4), **(c)** Hp(0,4,4), and **(d)** Hp(0,10,4) at applied force of 2.0 nN subtracted by baseline value, showing no notable difference between different backbone bond types.

References

(1) Hahmann, J.; Schüpp, B. N.; Ishaqat, A.; Selvakumar, A.; Göstl, R.; Gräter, F.; Herrmann, A. *Chem* **2025**, *11*, 102376.

(2) Bohlin, J.; Matthies, M.; Poppleton, E.; Procyk, J.; Mallya, A.; Yan, H.; Šulc, P. *Nat. Protoc.* **2022**, *17*, 1762–1788.

(3) Suma, A.; Poppleton, E.; Matthies, M.; Šulc, P.; Romano, F.; Louis, A. A.; Doye, J. P.; Micheletti, C.; Rovigatti, L. *J. Comput. Chem.* **2019**, *40*, 2586–2595.

(4) Zgarbová, M.; Sponer, J.; Jurecka, P. *J. Chem. Theory Comput.* **2021**, *17*, 6292–6301.

(5) Berendsen, H.; Hess, B.; Lindahl, E.; Van Der Spoel, D.; Mark, A.; Groenhof, G. *J. Comput. Chem.* **2005**, *26*, 1701–1718.

(6) Grokhovsky, S. L.; Il'Icheva, I. A.; Nechipurenko, D. Y.; Golovkin, M. V.; Panchenko, L. A.; Polozov, R. V.; Nechipurenko, Y. D. *Biophys. J.* **2011**, *100*, 117–125.

(7) Richards, O. C.; Boyer, P. *J. Mol. Biol.* **1965**, *11*, 327–340.
